# A Risk-Aware Spatial Deployment Framework Followed by Hierarchical Network Organization and Energy-Aware Performance Assessment

**DOI:** 10.3390/s26216689

**Published:** 2026-10-22

**Authors:** Milton Gussule, Md Tofael Ahmed, Masud Rana Rashel, Nuno Ribeiro, Fátima Baptista, Mouhaydine Tlemçani

**Affiliations:** 1 Departamento de Engenharia Rural, MED Mediterranean Institute for Agriculture, Environment and Development & CHANGE Global Change and Sustainability Institute, Universidade de Évora, 7006-554 Évora, Portugal; d39583@alunos.uevora.pt (M.G.); fb@uevora.pt (F.B.); 2 Instrumentation and Control Laboratory-LAICA, Center for Sci-Tech Research in Earth System and Energy-CREATE, University of Évora, 7000-671 Évora, Portugal; mrashel@uevora.pt (M.R.R.); tlem@uevora.pt (M.T.); 3 Center for Sci-Tech Research in Earth System and Energy-CREATE, University of Évora, 7000-671 Évora, Portugal; nmcar@uevora.pt

**Keywords:** wireless sensor networks, sensor placement, early detection of forest fires, hazard mapping, geographic information systems, coverage optimization, energy efficiency, clustering

## Abstract

**Highlights:**

This section summarizes, in bullet points, the main scientific and practical contributions of the study.

**What are the main findings?**

- An integrated sensor placement methodology directly uses the official fire hazard mapping of the ICNF to guide the deployment of WSN in the Serra de São Mamede Natural Park.
- The greedy MCP selected 589 sensor positions to cover 2995 priority cells classified as high and very high hazard.
- With the same number of sensing nodes (589), the combined hazard-prioritized, coverage-optimized placement covered 100% of the high- and very-high-hazard cells, whereas random placement covered only 45.3 ± 1.5% (mean ± SD over 100 random placements), for a similar estimated energy consumption per round (349.6 J versus 339.4 J).
- Under a conservative 250 m sensor-to-sensor range, multi-hop connectivity required about two relay nodes per sensor (1159–1174 relays), and the energy differences among 28, 29 and 30 clusters were small (≤1.8%) and are reported descriptively.

**What are the implications of the main findings?**

- The integration of hazard mapping, GIS, and WSN optimization allow monitoring resources to be directed to spatially prioritized areas.
- The methodology provides a reproducible framework for planning early fire detection networks in territories with available official hazard mapping.
- The approach can be adapted to different study areas and supplemented with dynamic environmental information to support spatially risk-oriented monitoring systems.

**Abstract:**

Forest fires are a growing threat in Portugal and in the Mediterranean region, necessitating the development of early detection systems capable of deploying monitoring resources to the highest priority areas. Conventional strategies for sensor placement in WSNs are often based on coverage, distance, and connectivity criteria, without explicitly incorporating the spatial distribution of fire hazard. This study proposes an integrated methodology for risk-oriented WSN planning, using the ICNF’s official rural fire hazard map as a spatial decision criterion. The cartography is converted into a hazard matrix and used to identify priority areas, on which the maximum coverage problem (MCP) was applied through a greedy heuristic to determine the sensor deployment positions. The resulting network was subsequently organized using K-means, with cluster head (CH) selection, relay-assisted multi-hop routing under a 250 m communication range, sink identification, and inter-cluster communication via a minimum spanning tree (MST), interpreted as an idealized connected graph and also recomputed under the same 250 m link constraint. Two alternative scenarios were also evaluated: a less restrictive hazard threshold (classes 3–5) and a random deployment of the same number of sensing nodes, repeated over 100 independent seeds, and a hazard-blind coverage-optimized control. The methodology is applied to the Serra de São Mamede Natural Park, with an area of 56,061.31 ha, where 2995 priority cells classified as high and very high hazard is identified. The greedy heuristic placed 589 sensors that covered all priority cells, whereas random deployments of the same 589 sensing nodes covered only 45.3 ± 1.5% of them (a hazard-blind optimized deployment covered 61.1%), for a similar estimated energy consumption (349.6 J versus 339.4 J per round), which corresponds to about half the energy per covered priority cell; this gain reflects the combined effect of hazard prioritization and coverage optimization. Connecting the sensors under the 250 m range required 1159–1174 relay nodes, and the energy differences among 28–30 clusters were small (≤1.8%) and are treated as descriptive. Extending the priority set to classes 3–5 required 1054 sensors and, with the cluster count held fixed at k = 29, increased energy consumption by 70.6% (21% with k = 53, i.e., about 20 sensors per cluster). The results indicate that the integration of official hazard mapping, spatial optimization, hierarchical organization, and routing constitutes a reproducible approach for risk-oriented WSN planning, with the potential to support early fire detection systems in spatially heterogeneous territories.

## 1. Introduction

Forest fires pose a significant threat to ecosystems, biodiversity, and natural resources, with increasingly severe environmental, social, and economic consequences. According to the European Forest Fire Information System (EFFIS), in 2022 fires were recorded in 45 European countries, with Portugal among the five most affected countries, with 112,063 hectares of burned area [1]. In 2024, the burned area in Portugal increased by 22% compared to the average of the previous ten years [2]. This situation worsened in 2025, the year in which the European Union recorded the most severe fire season on record, with more than one million hectares burned. In Portugal, 999 fires were recorded, responsible for 284,012 hectares of burned area, approximately double the area burned in the previous year, of which more than 51,000 hectares occurred in protected areas [3].

Despite the high severity of recent fire seasons, the total number of fire incidents in Portugal has shown a downward trend. In 2025, there were approximately 8252 rural fires, corresponding to the third lowest value since 2001. However, only 44 occurrences were responsible for approximately 91% of the total area burned in the country [4]. This relationship highlights a characteristic that is particularly relevant for fire risk management, namely, the frequency of ignitions does not necessarily reflect the magnitude of the impact. A small proportion of occurrences can, however, evolve into large-scale and highly severe fires. Thus, monitoring should not only seek to maximize the area generically covered but also ensure a greater detection capacity in areas where the probability of occurrence and the potential for fire spread are higher.

The occurrence and spread of fires result from the interaction between meteorological conditions, topographic characteristics and properties of plant fuel [5]. In Portugal, factors such as high density and continuity of vegetation, rugged terrain, and Mediterranean climatic conditions contribute to a high spatial heterogeneity of fire risk [6,7,8,9,10]. Additionally, the abandonment of traditional agricultural and forestry practices can favor the accumulation of combustible biomass, increasing fuel availability in certain areas [9,10]. The causes of ignition are also strongly associated with human activity, highlighting arson and different forms of fire use in agricultural and forestry activities [11,12,13,14]. Regardless of the origin of the ignition, the subsequent evolution of the fire depends on the environmental conditions existing at the site and time of occurrence. Consequently, fire risk presents a heterogeneous spatial distribution, making the adoption of monitoring strategies capable of differentiating areas according to their risk level relevant.

The prevention, monitoring, and detection of fires have benefited from significant advances in technologies such as remote sensing, unmanned aerial vehicles (UAVs), computer vision, artificial intelligence, and machine learning [15,16,17,18,19]. Despite their advantages in monitoring large areas, these approaches may present limitations related to temporal resolution, atmospheric conditions, availability of communication infrastructure, and the ability to identify incipient signs of fire [19,20,21]. Recent reviews also highlight challenges related to the generalization of models across different ecosystems, real-time data integration, and reliable detection of events in early stages [22]. These limitations reinforce the need for complementary approaches capable of performing continuous and localized monitoring directly in the field.

Wireless Sensor Networks (WSNs) represent a promising approach for this type of monitoring, enabling the distributed and continuous acquisition of environmental variables in extensive and potentially remote areas [23]. A WSN can integrate multiple sensor nodes responsible for collecting environmental parameters and transmitting the data through a distributed communication infrastructure. This architecture allows for high-resolution spatial and temporal monitoring and may be particularly suitable for forest environments where the installation of conventional infrastructure is limited. However, the effectiveness of a WSN depends heavily on the location of its sensor nodes. Inadequate distribution can leave critical zones uncovered, concentrate monitoring resources in lower-priority areas, or increase communication distances and, consequently, the network’s energy consumption.

In this context, the positioning of sensor nodes constitutes a spatial optimization problem that involves a trade-off between coverage, positioning, location, connectivity, and efficient use of energy resources. A significant portion of the literature has addressed this problem by maximizing geometric coverage, using random strategies, regular grids, or metaheuristic algorithms. In [24], for example, a particle swarm optimization algorithm was proposed to improve the continuous coverage of a WSN. In [25], a coverage model was proposed that considers path loss and false alarm probability, while [26,27] developed a deployment optimization strategy based on the Desert Golden Mole Optimization Algorithm. In the specific context of forest fire monitoring [28] used a modified version of the Marine Predator Algorithm to optimize the coverage and distribution of sensor nodes. In parallel, Geographic Information Systems-based approaches have demonstrated the usefulness of spatial risk information to support territorial planning decisions, as in [29], which combines fire risk mapping and UAV deployment planning. More recently, [30] integrated spatial patterns of fire occurrence derived from satellite data into a multi-objective WSN deployment optimization problem, formulating sensor coordinates as continuous decision variables and simultaneously considering coverage, connectivity, and energy consumption. Complementarily, [31] proposed a dynamic fire simulation and multi-objective optimization-based approach to determine robust sensor configurations in the face of different ignition probabilities and weather conditions.

Recent literature demonstrates an evolution in sensor network deployment strategies, from approaches focused predominantly on geometric coverage and the network’s perception and communication characteristics to formulations that incorporate spatial information on fire risk and behavior. While some studies seek to optimize network coverage or perception characteristics [24,25,26,27,28], more recent work has explored the use of spatial risk information, fire behavior simulation, and multi-objective optimization to guide sensor placement [29,30,31]. This evolution demonstrates the relevance of deployment strategies adapted to the spatial and dynamic characteristics of environments subject to forest fires.

Despite these advances, the integration between a discretized representation of fire risk, spatial prioritization of coverage, and hierarchical network organization remains insufficiently explored. In particular, there is room for approaches that transform a spatial risk map into a finite structure of decision units, allowing the prioritization of different zones to be incorporated into the sensor site selection process and subsequently articulated with cluster formation, CH selection, and energy consumption assessment. This approach is particularly relevant in scenarios where the number of available sensors is limited, and a uniform distribution of nodes can lead to inefficient use of monitoring resources.

In this context, this study proposes a discretized representation of risk in spatial decision units, integrating this prioritization with a coverage model and a hierarchical architecture based on clusters and Cluster Heads (CHs), simultaneously evaluating the trade-off between risk-oriented coverage and energy efficiency.

The main objective of this study is to develop and evaluate a risk-oriented positioning strategy for a hierarchical Wireless Sensor Network intended for the early detection of forest fires. The approach integrates spatial information associated with fire risk into a discretized representation of the territory, using the different spatial units as a basis for establishing coverage priorities and guiding the selection of sensor deployment locations. The resulting configuration is subsequently organized into a hierarchical architecture based on clusters and Cluster Heads (CHs), and evaluated in terms of coverage, connectivity, and energy consumption. The main contributions of this study are:

1. Discretized spatial formulation of the WSN deployment problem, in which a fire risk map is converted into a regular grid of spatial units classified according to risk level;
2. Risk-oriented positioning model, which uses the priority of spatial units to direct monitoring resources to higher-risk zones, instead of exclusively optimizing overall geometric coverage;
3. Integration of risk-oriented positioning into a hierarchical WSN architecture, including cluster formation and selection of Cluster Heads;
4. Comparative evaluation of network configurations in terms of coverage, connectivity, and energy consumption, allowing analysis of the trade-off between spatial monitoring priority and communication.

The remainder of the article is organized as follows. Section 2 presents the work related to forest fire detection based on WSNs and sensor positioning and optimization strategies. Section 3 describes the proposed methodology, including the construction and discretization of the risk map, definition of spatial units, coverage model, sensor positioning, network connectivity, cluster formation, and selection of Cluster Heads. Section 4 presents the results and comparative evaluation of different network configurations, with particular focus on coverage and energy consumption. Finally, Section 5 presents the main conclusions, the limitations of the study, and proposals for future work.

## 2. Related Works

### 2.1. Fire Detection Based on WSN

Several studies have explored WSNs for early detection and monitoring of forest fires. Recent reviews highlight the potential of IoT-based and WSN-based terrestrial solutions as a complement to remote sensing systems, particularly for their ability to perform continuous and localized monitoring and reduce response time [32]. WSN-based architectures allow for the distributed collection of environmental variables, such as temperature, humidity, and gases, and the transmission of alerts via wireless communication networks [33,34,35,36,37]. Different implementations have resorted to low-cost platforms, such as Arduino and NodeMCU, and long-range communication technologies, including LoRa, to enable the installation of sensors in remote environments [15,37,38,39,40].

Research has also sought to increase the robustness of WSNs through the use of adaptive communication and processing strategies. In [41], for example, the authors propose a cognitive radio sensor network with adaptive priority management, seeking to improve alert transmission in situations of congestion or unstable connectivity. Other approaches combine physical sensors with intelligent models to anticipate conditions associated with fire development [21]. Taken together, these works demonstrate the ability of WSNs to support distributed monitoring and early detection, although system performance depends heavily on the spatial distribution of the nodes.

The versatility of WSNs is further evidenced in applications such as ecosystem monitoring [42] and the detection of forest fires in remote and challenging scenarios [43,44,45], using image processing [46], long-range radios [37] and sound alert systems for wildlife protection [47]. This diversity of applications has been validated in real implementation conditions. In a study published in Scientific Reports [48], the practical feasibility of a WSN combined with machine learning for forest fire detection was demonstrated, showing improvements in accuracy and response time compared to conventional surveillance methods.

### 2.2. Environmental Parameters and Detection

Monitoring environmental parameters is a fundamental component of early detection systems for forest fires. Variables such as temperature, relative humidity, and gas concentration can be used to identify changes in environmental conditions associated with the occurrence or evolution of fires [49,50,51,52,53]. These measurements are often complemented by meteorological variables, including wind speed and direction, and atmospheric pressure, allowing for a more comprehensive characterization of surrounding conditions [54,55,56], as well as by low-cost thermal sensors that constitute an economically viable alternative to FLIR thermal cameras in distributed monitoring applications [57].

Thermal sensors and low-power gas sensors have been particularly studied due to their suitability for distributed networks, where energy consumption is a significant constraint [45,46,47,48,49,50,51,52,53,58,59,60,61]. The use of multiple sensor types also allows for combining different sources of evidence and reducing dependence on a single detection variable.

Although these approaches contribute to increasing the ability to identify fire signals, their effectiveness depends not only on the characteristics of the sensors, but also on the location of the nodes and the network’s ability to adequately cover the monitored territory.

### 2.3. Virtual Sensors and Complementary Detection

In addition to physical sensors, different studies have explored complementary sources of information, including acoustic detection and virtual sensors. Acoustic detection is based on the identification of sound patterns associated with combustion and flame propagation, often using signal processing and machine learning techniques [62,63,64,65,66,67,68,69,70]. Despite promising results, the performance of these systems can be affected by anthropogenic noise and adverse environmental conditions.

Virtual sensors constitute another complementary approach, allowing the estimation of variables that are not directly measured through computational models fed by data from physical sensors [71,72,73,74,75,76,77,78] and strategies for the safe positioning of resources in virtual networks [79]. These techniques can contribute to compensating for communication failures and reducing information gaps, especially in remote areas.

However, both physical and virtual sensors depend on the quality and availability of the information collected by the network. Thus, the spatial distribution of sensors continues to be a determining factor for the overall coverage and reliability of the system.

### 2.4. Factors Affecting Detection Accuracy

The performance of early detection systems is conditioned not only by the technologies used, but also by the environmental and physical characteristics of the territory. Factors such as fuel type, terrain slope, and wind speed influence fire spread and the time available for its detection and response [80,81]. The occurrence of false positives is also a challenge, and may be associated with weather conditions, air pollution, or limitations of the sensors used [54,82,83].

The physical characteristics of the territory can also affect communication between nodes and network coverage. Mountainous areas and regions with high vegetation density can hinder signal propagation and increase areas without adequate coverage [84]. Reducing the number of sensors tends, in turn, to increase detection time and decrease monitoring reliability [85], although multisensor strategies can help mitigate these limitations [86]. The slope of the terrain, the density of vegetation cover and other environmental factors directly influence the accuracy of early detection models, reinforcing the need to integrate this information into the planning and operation of WSNs [87].

These results show that the effectiveness of a WSN does not depend exclusively on the individual capacity of the sensors. The spatial distribution of the nodes is also a fundamental element to ensure that monitoring resources are placed in areas where they can produce the greatest benefit.

### 2.5. Sensor Positioning and Optimization

The location of sensor nodes is one of the main factors determining the coverage, connectivity, and energy efficiency of WSNs applied to forest fire detection. Inadequate distribution can produce uncovered zones, redundancy between sensors, and unnecessarily high communication distances, simultaneously compromising detection capacity and network lifespan [37,88,89,90,91].

Early positioning approaches generally resort to random distributions, regular grids, or geometric models based on coverage and connectivity. Although these strategies allow for structuring network deployment, they generally treat the territory as a homogeneous space, in which different positions have equivalent importance. This assumption may be inadequate in forest environments, where the fire risk has a heterogeneous spatial distribution.

To overcome these limitations, different optimization methods have been proposed to determine the location of the nodes. These include mathematical formulations aimed at maximizing coverage and minimizing overlaps, as well as bi-objective models that simultaneously consider cost and coverage objectives [92], integer programming [93] and strategies oriented towards energy efficiency and connectivity [94]. Metaheuristic algorithms have also assumed an important role in this domain, including Genetic Algorithms [95], Desert Golden Mole Optimization Algorithm [26], Arithmetic Optimization Algorithm [96] and different variants of Particle Swarm Optimization [24]. Other works combine different metaheuristics to simultaneously improve network coverage and energy use [97,98].

Despite the advances achieved, these strategies often formulate positioning as a problem of geometric coverage, connectivity or node distribution, seeking to find an efficient configuration for the monitoring space considered. In these formulations, a covered area generally has a similar value regardless of its location. However, in the context of forest fires, different spatial units may present distinct risk levels and, consequently, different monitoring priorities.

This issue has motivated more recent approaches aimed at incorporating spatial information into the deployment process. A Fire Risk Index (FRI) calculated from environmental variables collected by sensors is proposed in [99], allowing support for dynamic risk monitoring. In another approach [100], the network topology and density are adjusted according to structural characteristics of the forest, distinguishing different relief conditions and vegetation density.

A particularly relevant example of integration between spatial fire information and WSN planning is presented in [30], using historical fire detection data obtained using the NASA FIRMS VIIRS system to guide the optimization of sensor location.

Complementarily, an approach based on dynamic simulation of fire behavior to optimize the configuration of sensor networks was proposed in [31], considering different ignition probabilities and climatic conditions. The multi-objective formulation seeks to obtain robust configurations in the face of different fire scenarios, highlighting the importance of considering the spatial and temporal variability of the phenomenon in the definition of the network.

Although these recent works represent important advances in the integration between spatial risk information and sensor positioning, the approaches considered predominantly formulate the sensor coordinates as decision variables in a continuous space, resorting to optimization processes to determine the final network configuration [30,31]. It therefore remains relevant to investigate a formulation in which discrete spatial units directly constitute the decision-making units to establish coverage priorities and select deployment locations.

Other studies, however, continue to favor models based exclusively or predominantly on geometric coverage, including strategies based on k-coverage [101,102], geographic density [103,104], Voronoi diagrams [105,106] and deployment strategies oriented towards the target coverage problem [107]. These studies are relevant to the formulation of the coverage problem but represent the territory essentially in geometric terms and do not necessarily establish an explicit relationship between the spatial priority associated with risk and the selection of deployment sites.

### 2.6. Energy Efficiency and Hierarchical Network Organization

Energy efficiency is a key factor in the long-term operation of wireless sensor networks, particularly in remote forest environments where replacing or recharging sensor node batteries can be difficult. Energy consumption is associated with the different operations performed by the nodes, including data acquisition and processing, but it is particularly relevant during communication due to the energy required for transmitting and receiving information [108,109,110,111]. In this context, several strategies have been proposed to extend the lifespan of networks, including node activity control mechanisms, coverage optimization, and energy-efficient routing protocols [108,109,110,111,112].

The hierarchical organization of the network through the formation of clusters is one of the strategies used to reduce the cost of communication. The LEACH (Low-Energy Adaptive Clustering Hierarchy) protocol, proposed by Heinzelman et al. [113] constitutes a fundamental reference in this domain, by organizing the sensor nodes into clusters and selecting, in each group, a CH responsible for aggregating and forwarding the data. This structure allows reducing the number of direct communications between the sensor nodes and the base station, distributing the communication functions among the different elements of the network [113]. Based on this principle, several variants of clustering protocols have been developed with the aim of improving the distribution of energy consumption and extending the lifespan of the network.

The selection of Cluster Heads is, in this context, a particularly relevant step. The CHs assume additional functions of receiving, aggregating and transmitting data, and may, consequently, have a higher energy consumption than the other nodes. Inadequate selection can concentrate traffic on certain sensors and accelerate the depletion of their batteries, compromising the connectivity and lifespan of the network [114,115,116]. For this reason, different approaches have sought to select CHs considering factors such as residual node energy, communication distance, network density, and spatial distribution of sensors [113,117,118,119]. Evolutionary optimization methods and fuzzy logic-based techniques have also been applied to the dynamic selection of CHs with the aim of balancing energy consumption and improving data routing [118,119].

The relationship between hierarchical organization and energy efficiency is of particular importance in forest fire detection applications. In these applications, the occurrence of a fire can cause a significant increase in the frequency of data transmission, causing the nodes involved in the detection and routing of information to have a higher consumption than that observed during conventional monitoring. It has been demonstrated that the use of a cluster-based architecture can improve the energy efficiency of fire monitoring [120], while an energy-efficient clustering approach specifically oriented towards the early detection of forest fires was proposed in [117]. Other works have also explored IoT architectures based on WSN for fire detection, highlighting the importance of communication efficiency and the availability of energy from the nodes [121].

In forest environments, however, energy efficiency cannot be analyzed independently of the spatial configuration of the network. The distance between nodes influences the cost of transmission and, consequently, the energy consumed in communication. Additionally, specific characteristics of the forest environment, such as relief, vegetation, and three-dimensional forest structure, can affect signal propagation and cause communication attenuation. Recent studies in three-dimensional forest scenarios, therefore, have simultaneously considered terrain, vegetation, CH selection, and energy routing of data [122]. These characteristics reinforce the need to evaluate energy efficiency considering the spatial configuration of the sensors and the resulting communication conditions.

In this context, cluster formation and CH selection constitute a complementary step to sensor placement. While risk-oriented placement determines where sensors should be installed, assigning higher priority to spatial units associated with higher risk levels, hierarchical organization determines how these sensors can communicate efficiently. This distinction is particularly important in the present study, since a spatial configuration suitable for maximizing coverage of the highest-risk zones does not necessarily imply an energy-efficient communication organization.

Thus, energy efficiency is considered, in the present study, as a complementary component of the deployment strategy. Initially, spatial risk information guides the selection of units where sensors should be positioned. Subsequently, the sensors resulting from this configuration are organized into clusters, and the respective Cluster Heads are selected, allowing for the evaluation of the energy implications of the communication organization. This approach makes it possible to jointly analyze the adequacy of coverage for areas of greatest risk and the energy consumption associated with network communication, providing a more comprehensive assessment of the proposed configuration.

## 3. Materials and Methods

This study proposes an integrated methodology for planning and organizing Wireless Sensor Networks for the early detection of forest fires. The approach combines official geospatial information, Geographic Information Systems (GIS) techniques, spatial optimization, and hierarchical network organization. Unlike approaches that treat sensor placement predominantly as a geometric problem, the proposed methodology uses the official fire hazard mapping provided by the Institute for Nature Conservation and Forests (ICNF) as the main criterion to guide sensor location.

**Figure 1:**
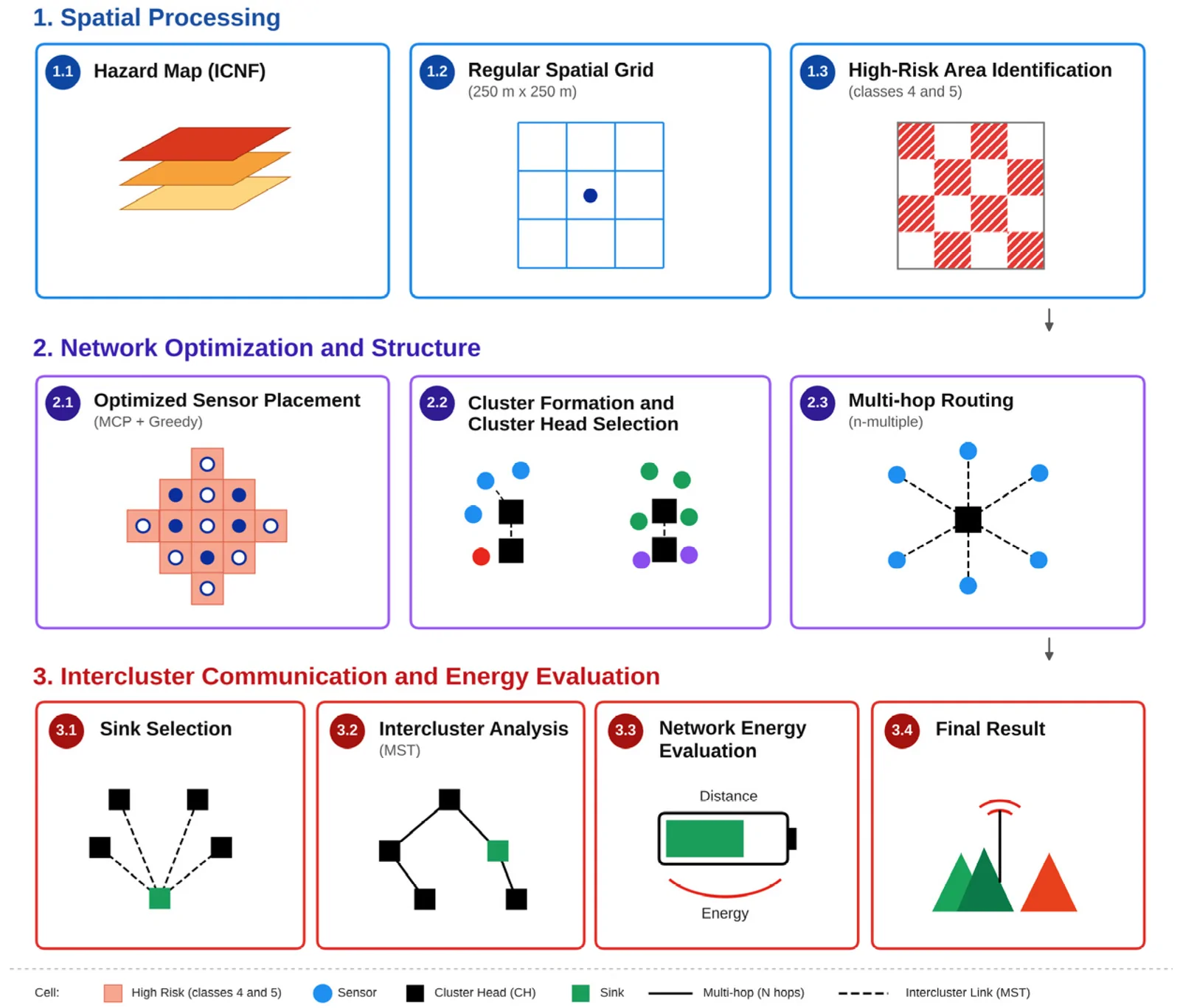
Flowchart summarizing the main steps of the proposed methodology. In panel 2.2, dot colours distinguish the sensors of different clusters; all other symbols are explained in the legend at the bottom of the figure.

The methodology is structured in sequential and interdependent steps (Figure 1). First, the hazard map was processed and converted into a regular matrix representation of the study area (Section 3.1 and Section 3.2). Second, priority cells for monitoring were identified according to a hazard threshold. Three hazard-selection scenarios were defined to evaluate the influence of this threshold (Section 3.3). Third, sensor positions were determined with a greedy coverage heuristic. A random deployment with the same number of sensors served as a control (Section 3.4). The resulting network was then organized into clusters using the K-means algorithm, and the respective Cluster Heads (CHs) were defined. Relay nodes were added where necessary to guarantee multi-hop connectivity between sensors and their CHs within the assumed communication range. Intra-cluster communication was modelled with multi-hop routing, and inter-cluster communication was organized through a Minimum Spanning Tree (MST), with one CH acting as the sink. Finally, the energy consumption of each configuration was estimated using the first-order radio model of Heinzelman et al. [113].

Geospatial processing was performed in ArcGIS Desktop (ArcMap version 10.8, Esri, Redlands, CA, USA), which was used for the preparation and analysis of cartographic data. The R environment (R Foundation for Statistical Computing, Vienna, Austria), run in RStudio (version 2024.12.0+467; Posit Software, PBC, Boston, MA, USA), was used to implement the matrix processing procedures. Python 3.12 (Python Software Foundation, Wilmington, DE, USA) was used to identify priority areas and optimize sensor placement, analyze network topology, construct the MST, perform spatial visualization, and calculate energy consumption. Figure 1 presents a flowchart summarizing the main steps of the proposed methodology.

### 3.1. Spatial Hazard Mapping and Representation

**Figure 2:**
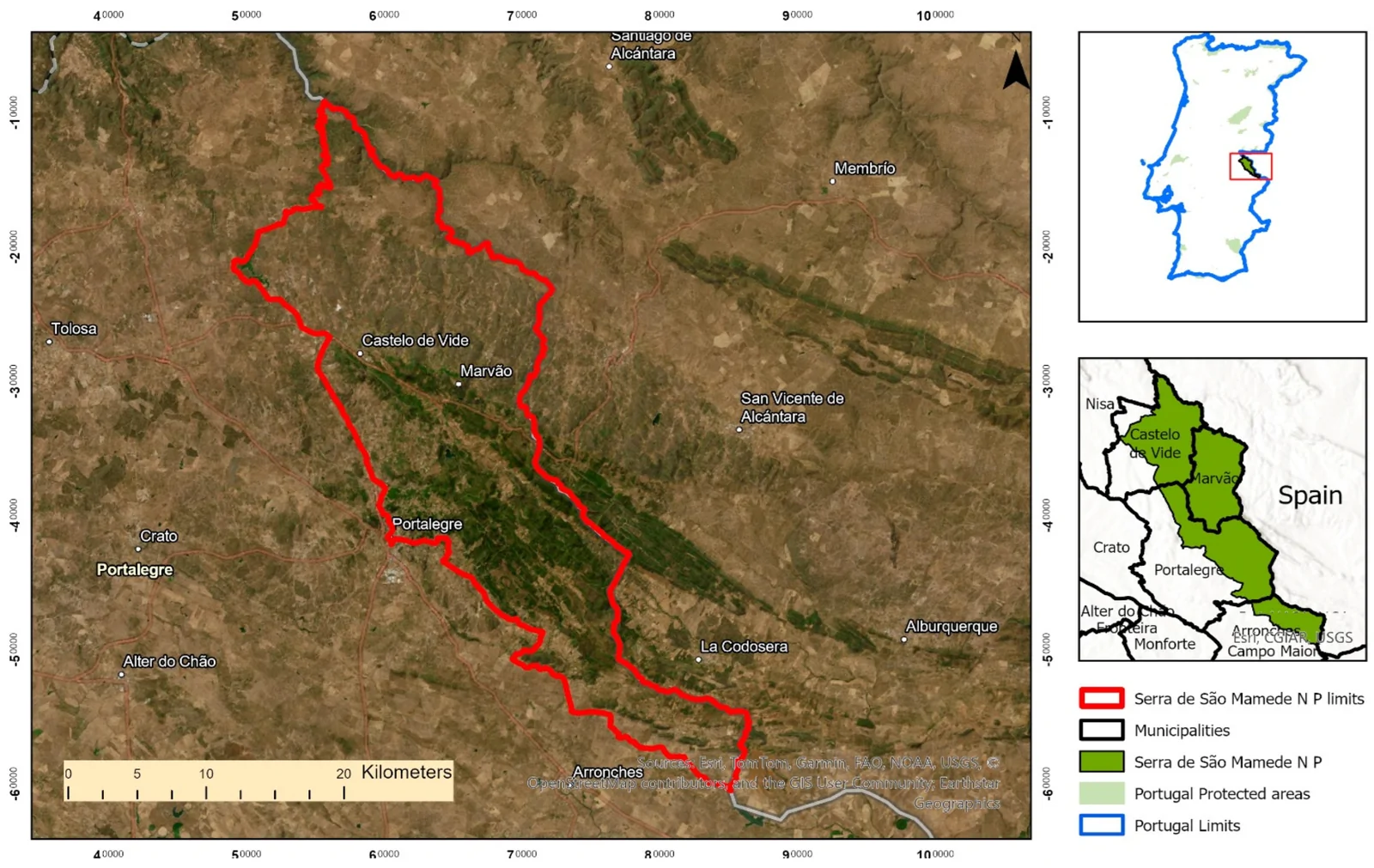
Location of the Serra de São Mamede Natural Park (PNSSM). Left: satellite image of the study area with the PNSSM boundary in red and the main settlements. Top right: mainland Portugal (blue outline) showing protected areas in light green, with the study area highlighted by the red rectangle. Bottom right: PNSSM (green) and municipal boundaries (black lines) along the border with Spain.

The study area is the Serra de São Mamede Natural Park (PNSSM), located in northern Alentejo, Portugal, and covering approximately 56,061.31 ha (Figure 2). The park extends across the municipalities of Arronches, Castelo de Vide, Marvão and Portalegre and constitutes the main mountain system in the region. Its steep relief, diverse vegetation cover and environmental conditions favorable to fire spread make the PNSSM a suitable area for evaluating forest fire detection strategies.

Hazard was characterized using the Official Rural Fire Hazard Map provided by the ICNF, which was the main source of spatial information on the distribution of fire hazard in the study area. In ArcMap, using the Spatial Analyst extension, the hazard map was converted to raster format and reclassified with the Reclassify tool into five discrete classes, numbered 1 to 5, corresponding to increasing hazard levels from very low to very high. This standardization represented hazard on a homogeneous numerical scale compatible with the subsequent computational steps.

### 3.2. Construction of the Regular Spatial Grid

**Figure 3:**
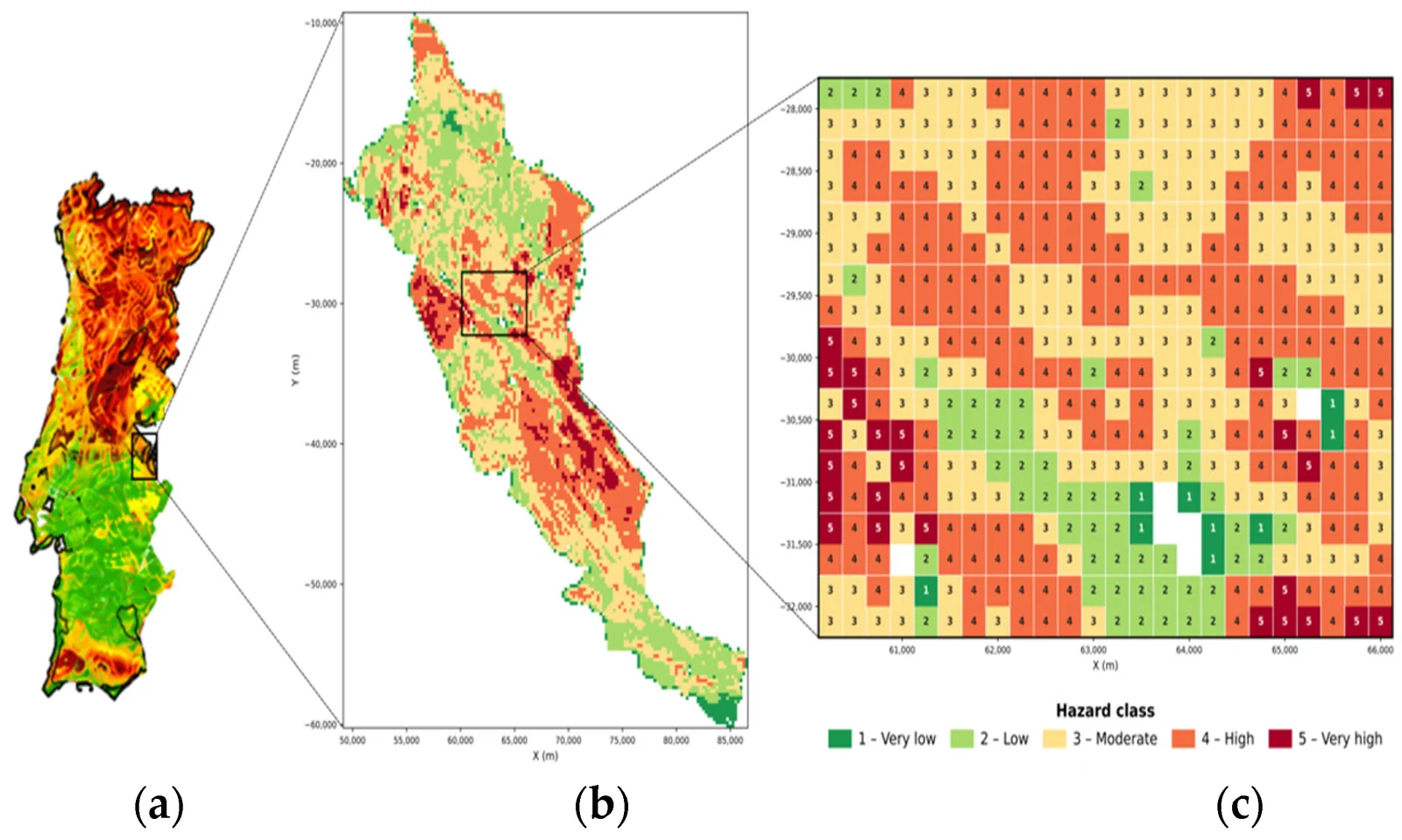
Spatial discretization of the hazard map into a 250 × 250 m grid. (**a**) PNSSM location; (**b**) fishnet grid over the hazard raster; (**c**) detail of classified cells with centroids. The black box in (**b**) marks the area enlarged in (**c**); colours indicate the hazard classes 1–5 listed in the legend.

A regular spatial mesh (fishnet) of 250 × 250 m square cells was then constructed over the entire study area, providing a uniform spatial discretization of the territory (Figure 3).

The cell size was chosen to match the operational range assumed for LoRa (Long Range) technology in the communication model. LoRa was selected for its low power consumption, robustness and long-range communication capability, which make it suitable for environmental monitoring in remote areas [123,124,125]. An operational range of approximately 250 m was assumed for forest environments. This is a conservative modelling assumption, since greater distances can be achieved under favorable conditions, particularly in open areas [126]. The 250 m resolution therefore establishes a consistent spatial relationship between the hazard representation and the sensor range. It also balances spatial resolution, representation of hazard heterogeneity and computational cost. Since the sensor placement does not guarantee that sensors lie within this range of one another, relay nodes were introduced where required.

Each cell was represented by its geometric center, with associated planimetric coordinates (X, Y). The hazard value of the raster was extracted at each cell center, so that every grid position was linked to its hazard level. Cells without a hazard class, including those outside the park boundary, were assigned code 0.

The resulting dataset, containing the coordinates (X, Y) and the hazard value of each cell, was processed in R and converted into a regular matrix, hereafter called the Numerical Hazard Map. In this representation, each matrix element corresponds to one grid cell and retains its geographic coordinates. The optimization algorithms can therefore operate directly on the matrix structure without losing the spatial reference of the territory.

### 3.3. Identification of Priority Cells and Definition of Scenarios

This step transforms the spatial hazard information into a discrete set of priority cells, which defines the area that the sensor network must cover. A Python routine traversed the Numerical Hazard Map and selected the cells whose hazard classes belonged to a predefined set. The hazard threshold was thus treated as an explicit parameter of the methodology, and three main scenarios were defined, complemented by two supplementary controls:

(i) a reference scenario, in which cells of Classes 4 and 5 (high and very high hazard) were considered priority cells, concentrating monitoring resources on the sectors most susceptible to fire;

(ii) an alternative scenario, in which Classes 3 to 5 were considered priority cells, to assess the effect of a less restrictive threshold;

(iii) a control scenario without hazard prioritization, in which all cells with any hazard class (1–5) were considered eligible for a random deployment;

(i-c) a common-domain variant of the reference scenario, with the same priority cells (Classes 4 and 5) as scenario (i), but with sensor positions restricted to the classified cells (Classes 1–5), that is, the same installation set as the control scenario (iii);

(iv) a hazard-blind, coverage-optimized control, in which all classified cells (Classes 1–5) were treated as equally important demand cells and covered with the same number of sensors and the same feasible positions as the control scenario (iii), but with positions selected by the coverage heuristic instead of random sampling.

Scenarios (i-c) and (iv) are described in Section 3.4.4. Together with scenario (iii), they make it possible to separate the effect of coverage optimization from that of hazard prioritization under a common feasible domain.

In all scenarios, cells with code 0 were excluded from the set of priority or eligible cells. The coordinates of the selected cells were linked to their respective matrix indices, ensuring alignment between geographic location and the matrix structure. For each scenario, the output of this step was a set of cells, with their coordinates and matrix indices, which served as input for the sensor allocation step.

### 3.4. Sensor Positioning

#### 3.4.1. Coverage Problem Formulation

The Maximum Coverage Problem (MCP) is a classical combinatorial optimization problem. Its objective is to select a set of installation positions that covers a predefined set of demand areas, subject to the spatial and deployment constraints of the system [127]. The MCP is NP-hard, so obtaining an exact optimal solution may become computationally impractical as the number of candidate positions and demand cells increases. For this reason, a greedy heuristic was adopted.

Let I be the set of candidate positions, consisting of all cells of the grid, including cells without hazard classification, so that sensors near the park boundary can cover priority cells from adjacent positions. This feasible installation set is larger than that of the random control, which is restricted to the 9043 classified cells (Section 3.4.3); the effect of this difference was quantified by repeating the optimization with the candidate positions restricted to the classified cells (Section 3.4.4). Let P be the set of priority (demand) cells defined in Section 3.3. Each sensor was assumed to monitor the cell in which it is installed and the priority cells within a monitoring range of r cells. In this study, r = 1; that is, each sensor covers its own cell and the eight surrounding cells, including the diagonal neighbors (a 3 × 3 Moore neighborhood). The center-to-center distance to the covered cells is therefore 250 m for orthogonal neighbors and approximately 354 m for diagonal neighbors. The coverage set of candidate position i is defined as:

N_i_ = { j ∈ P:max(|row_i_ − row_j_|, |col_i_ − col_j_|) ≤ r } (1)

Only cells belonging to *P* are counted towards coverage, and sensors located at the edge of the grid cover fewer cells. The binary decision variable is:

X*_i_* = 1, if a sensor is installed in the position i; x*_i_* = 0, otherwise.

#### 3.4.2. Greedy Heuristic

The heuristic builds the solution iteratively. Let U be the set of priority cells not yet covered in a given iteration. The marginal gain associated with candidate position i is calculated by:

G(i) = |N_i_ ∩ U| (2)

where N_i_ represents the set of priority cells potentially covered by position i.

In each iteration, all candidate positions are evaluated and the position with the highest marginal gain is selected:

i^*^ = arg max_iϵI_G(i) (3)

The selected position receives a sensor ($x_{i^{*}}=1$), and the cells it covers are removed from *U*. The procedure is repeated until all priority cells are covered (*U* = ∅) or no candidate position provides additional coverage. The total number of sensors is then $S=\sum_{i\in I}x_{i}$.

The number of sensors was therefore not defined a priori. It emerged from the iterative process and depended on the extent and spatial distribution of the priority cells in each scenario. The heuristic was not formulated as a multi-objective problem with an explicit trade-off between coverage and number of sensors. Its objective was complete coverage of the priority cells through the successive selection of the positions with the highest marginal gain. When iterated until full coverage is achieved, this procedure corresponds to the classical greedy algorithm for the set covering problem. As a result, sensors are concentrated where priority cells are located, rather than distributed uniformly across the study area. The greedy heuristic was applied to the reference (i) and alternative (ii) scenarios.

#### 3.4.3. Control Scenario: Random Deployment

In the control scenario (iii), sensor positions were not optimized. The same number of sensors *S* obtained in the reference scenario (i) was randomly placed among the eligible cells (hazard Classes 1–5), using uniform sampling without replacement, so that no cell received more than one sensor. The random deployment was repeated for *N_p_* = 100 independent seeds (Section 3.10); the draw with seed = 42 used for the single-run results is one realisation of this distribution. This equal sensing-node design ensures that differences between scenarios (i) and (iii) reflect the deployment strategy, hazard-based selection combined with coverage optimization versus non-prioritized random placement, rather than the number of sensing nodes. It is not an equal total-deployment-cost comparison: the number of relay nodes, by contrast, was not fixed, as it results from the spatial configuration of each scenario and constitutes one of the outcomes compared.

To compare the effectiveness of both deployment strategies, the coverage achieved in scenario (iii) was computed using the same coverage criterion as in the optimized scenarios (Equation (1)) and expressed as the percentage of class 4–5 cells (and class 3–5 cells) covered by at least one sensor.

#### 3.4.4. Common Feasible Domain and Hazard-Blind Control

The comparison between scenarios (i) and (iii) changes two factors at once: the set of cells that the deployment is intended to cover (hazard prioritization) and the way positions are chosen (coverage optimization versus random sampling). In addition, the optimized scenarios may install sensors in unclassified cells outside the park, whereas the random control may not. Two supplementary analyses were therefore carried out.

First, to place both methods on a common feasible domain, the greedy heuristic of the reference scenario was repeated with the candidate set I restricted to the 9043 classified cells, that is, the same installation set as the random control (scenario i-c). The number of sensors required, the coverage of Class 4–5 cells and the network metrics were compared with those of the unrestricted solution, and the number of sensors of the unrestricted solution located in unclassified cells was recorded.

Second, to separate the contribution of hazard prioritization from that of coverage optimization, a hazard-blind, coverage-optimized control (scenario iv) was evaluated. The greedy heuristic was applied with the same feasible positions as scenario (iii) (classified cells) and the same sensor budget S = 589, but all classified cells (Classes 1–5) were treated as equally important demand cells, so that the heuristic maximizes geometric coverage of the park without any hazard information; because the budget is fixed, the heuristic was stopped after S selections. Since many candidate positions share the same marginal gain when the budget is smaller than that required for full coverage, ties were broken at random, and the control was repeated for 20 tie-breaking seeds. Coverage of Class 4–5 cells, relay count and energy were then computed with the same procedure as in the other scenarios. The difference between scenarios (iii) and (iv) estimates the effect of coverage optimization alone, and the difference between scenarios (iv) and (i-c) estimates the additional effect of hazard prioritization under a common feasible domain and an equal number of sensing nodes.

#### 3.4.5. Contribution and Output of This Step

The methodological contribution of this step does not lie in the greedy heuristic itself, which is well established for coverage and facility-location problems. It lies in the heuristic’s integration with the official forest fire hazard map produced by the ICNF. Because the hazard classification defines the cells to be covered, the sensor allocation incorporates the spatial distribution of fire hazard rather than relying exclusively on geometric or connectivity criteria.

Comparing the three scenarios makes it possible to assess how the definition of the priority set affects the resulting sensor configuration. The comparison between scenarios (i) and (ii) shows how the hazard threshold influences the number and location of sensors. The comparison between scenarios (i) and (iii) shows the combined effect of hazard-based filtering and coverage optimization relative to a non-prioritized random deployment with the same number of sensors. The supplementary scenarios of Section 3.4.4 separate the two components of this combined effect.

The final set of sensor positions obtained for each scenario was the input for the hierarchical organization of the network, described in the following sections.

### 3.5. Cluster Formation and Cluster Head Definition in Strategic Forest Locations

After the sensor positions were defined, the network was organized hierarchically into clusters. The K-means algorithm was used for this purpose. It is one of the most widely used unsupervised clustering methods in WSNs because of its computational simplicity, fast execution and ability to form spatially compact clusters.

K-means was applied to the two-dimensional coordinates of the sensors obtained in Section 3.4. It partitions the set of sensors into k clusters by minimizing the sum of squared distances between each sensor and the centroid of its cluster:

min_C1,…,Ck_ Σ_j=1_^k^ Σ_si∈Cj_ ‖s_i_ − μ_j_‖^2^ (4)

where Cj represents cluster j, s_i_ represents the position of sensor i, and μ_j_ corresponds to the centroid of the cluster. The algorithm was run with 10 independent initializations (n_init = 10), retaining the solution with the lowest objective value, and with a fixed random seed (random_state = 42) to ensure reproducibility. Reproducibility of a single run does not, however, establish the stability of the resulting network metrics; K-means was therefore also repeated over independent seeds, as described in Section 3.10.

The number of clusters was set so that each cluster contained, on average, approximately 20 sensors. This criterion follows Xu, Sun and Xiao [85], who report that increasing the number of sensors per cluster can reduce throughput, increase energy consumption and make intra-cluster communication less stable. For the reference scenario, this criterion led to k = 29. To assess the sensitivity of the network organization to this parameter, configurations with k = 28 and k = 30 were also evaluated. In scenarios (ii) and (iii), the same value k = 29 was used, so that all scenarios were compared under the same clustering configuration. Because this choice retains the cluster count selected for 589 sensors rather than applying the criterion of approximately 20 sensors per cluster, the alternative scenario (1054 sensors) was additionally evaluated with k = 53 (1054/20 ≈ 52.7) as a supplementary check.

The centroid produced by K-means is a geometric position and does not necessarily coincide with a physical sensor. Therefore, the Cluster Head (CH) of each cluster was defined as the sensor of that cluster closest to its centroid:

CH_j_ = argmin_si∈Cj_ d(s_i_, μ_j_) (5)

where *CH_j_* is the Cluster Head of cluster *j* and *d*(⋅,⋅) is the Euclidean distance. This ensures that every CH corresponds to an existing sensor and that no virtual nodes are introduced in the routing and energy models.

The CHs receive data from the sensors of their cluster and forward it towards the sink. The network communication is therefore structured at two levels, intra-cluster communication, between sensors and their CH (Section 3.6), and inter-cluster communication, between CHs and the sink (Section 3.7 and Section 3.8).

### 3.6. Multi-Hop Routing and Connectivity Between Sensors

Once the clusters and their CHs were defined, intra-cluster communication was modelled using multi-hop routing. Forwarding data through intermediate nodes, instead of transmitting directly over long distances, is a widely adopted strategy to extend network lifetime and improve scalability [82,128,129,130].

The communication range between nodes was set to *R_c_* = 250 m, corresponding to one grid cell. Because distances are measured between cell centers, only sensors located in orthogonally adjacent cells (250 m) fall within this range, whereas diagonal neighbors (≈354 m) do not. *R_c_* is therefore distinct from the monitoring range r used in the coverage model (Section 3.4), which includes diagonal cells. For two nodes P_1_(x_1_, y_1_) and P_2_(x_2_, y_2_), the Euclidean distance is:

(6) $$d\left(P_{1},P_{2}\right)=\sqrt{{\left(x_{2}-x_{1}\right)}^{2}+{\left(y_{2}-y_{1}\right)}^{2}}$$

The coverage heuristic positions sensors to maximize coverage of the priority cells, not to ensure connectivity between them. As a result, sensors may be separated by distances greater than *R_c_*. To guarantee that every sensor can reach its CH using links no longer than *R_c_*, relay nodes were introduced. Relays have no sensing function and only forward data.

Relays were placed using a minimum spanning tree approach. For each cluster *C_j_*, an MST was built over its sensors, using the distance of Equation (6) as edge weight. For each MST edge (u, v) whose length *d_uv_* exceeds *R_c_*, *n_uv_* relays were placed at equal intervals along the segment between the two sensors:

(7) $$n_{\mathrm{uv}}=\left\lceil \frac{d_{\mathrm{uv}}}{R_{c}}\right\rceil -1,\;\;p_{r}=s_{u}+\frac{r}{n_{\mathrm{uv}}+1}\;\left(s_{v}-s_{u}\right),\;r=1,\ldots ,n_{\mathrm{uv}}$$

This ensures that no link between consecutive nodes exceeds *R_c_*. Placing relays along the edges of a minimum spanning tree (the “steinerised” MST) is a classical approximation for the problem of connecting sensors with a minimum number of relay nodes [131,132]. Relays were not restricted to grid cell centers.

Routes were then computed on the communication graph *G_j_* of each cluster. Its nodes are the sensors and relays of cluster *C_j_*, and an edge connects two nodes when their distance does not exceed *R_c_*. Each edge was weighted by the energy required to transmit and receive one packet over that link, according to the radio model of Section 3.9. The route of each sensor *s_i_* to its Cluster Head is the minimum-energy path:

(8) $$\pi_{i}^{*}=\arg\underset{\pi \in \Pi \left(s_{i},CH_{j}\right)}{\min}\sum_{\left(a,b\right)\in \pi }\left[E_{\mathrm{TX}}\left(k,d_{\mathrm{ab}}\right)+E_{\mathrm{RX}}\left(k\right)\right]\;$$

where *Π*(*s_i_*,*CH_j_*) is the set of paths in *G_j_* between *s_i_* and *CH_j_*. Routes were computed with Dijkstra’s algorithm. Because the MST with relays connects all nodes of the cluster through links no longer than *R_c_*, every sensor has at least one route to its CH by construction.

For each scenario, the number of relays was recorded as an indicator of the connectivity cost of the sensor configuration, together with the number of hops and the distance travelled along each route. These values were subsequently used in the energy model.

### 3.7. Identification of the Central Node (Sink)

After the Cluster Heads were defined, a central node of the network, the sink, was selected. The sink is responsible for receiving the data from all clusters. Its location affects the structure of inter-cluster communication, in particular the number of hops and the distances over which data must be relayed before reaching it.

The sink was selected using a geometric centrality criterion: it was defined as the CH with the smallest sum of Euclidean distances to all other CHs, that is, the medoid of the set of CHs. Let *C* = {*CH*_1_,*CH*_2_,…,*CH_k_*} be the set of cluster heads are defined in Section 3.5. For each *CH_i_*, the sum of distances to the remaining CHs is

S(CH_i_) = Σ_j_≠_i_ d(CH_i_, CH_j_) (9)

where d(CH_i_, CH_j_) is the Euclidean distance (Equation (6)) between two Cluster Heads. The sink is then defined as:

Sink = argmin_{CH_i_ ∈ C} S(CH_i_) (10)

Selecting a spatially central CH reduces the overall separation between the sink and the remaining CHs. This tends to shorten the inter-cluster paths and the number of hops required for data to reach the sink, which in turn reduces communication latency. Inter-cluster data are routed towards the sink along the Minimum Spanning Tree described in the following section. The number of hops between each CH and the sink along this tree was recorded to characterize the resulting network structure.

### 3.8. Inter-Cluster Communication Structure (Minimum Spanning Tree)

Inter-cluster communication was organized through a Minimum Spanning Tree (MST) whose nodes are the Cluster Heads defined in Section 3.5. The weight of the link between two CHs was given by their Euclidean distance:

wij = d(CHi, CHj)(11)

The MST was obtained with Kruskal’s algorithm [133,134], as implemented in the minimum_spanning_tree function of the SciPy library [135]. The MST connects all CHs with the minimum total link length, guaranteeing connectivity of the structure and the absence of cycles.

Unlike the sensor-to-sensor and relay links, which are limited to *R_c_* = 250 m (Section 3.6), no range limit was imposed on the CH-to-CH links of the MST in its basic form. No hardware, antenna, installation or radio-configuration difference between CHs and ordinary sensors is assumed in this study: CHs are ordinary sensors selected by Equation (5), and nothing in the model gives them a longer range. Although LoRa links of several kilometres have been reported under favourable, line-of-sight conditions [126], such ranges are not substantiated for the forested and mountainous terrain of the PNSSM, and the longest MST links (up to 9361 m) cannot be considered feasible on this basis. The unconstrained MST is therefore interpreted only as an idealized connected graph, it establishes mathematical connectivity among the CHs, not the feasibility of the corresponding radio links. To obtain a physically consistent topology, the inter-cluster structure was also recomputed under the same link constraint as the intra-cluster layer (link-constrained variant), every MST edge longer than 250 m was subdivided by relay nodes according to Equation (7), and the inter-cluster energy was computed hop by hop with Equation (15) applied to the resulting links. Both variants are reported (Section 4.7 and Section 4.10.3).

The sink selected in Section 3.7 was used as the root of the tree, and all links were oriented towards it, defining a routing sequence between CHs. It was further assumed that each CH aggregates the data received from its cluster and from its child CHs before retransmission, reducing redundancy in the information transported through the network [136]. Under this assumption, every CH except the sink transmits exactly one aggregated packet to its parent node, so each MST link is used once per data-collection round. This simplification represents environmental monitoring systems in which multiple sensor readings can be combined before being forwarded to higher levels of the network.

Although the MST is built from distances, it also minimizes the transmission energy of the inter-cluster structure under the adopted model. A minimum spanning tree depends only on the ordering of edge weights. The tree that minimizes the sum of link lengths therefore also minimizes the sum of any increasing function of length, such as the distance-dependent amplifier term of the radio model presented in Section 3.9. With one aggregated packet per link, the MST is thus also the spanning tree with minimum total transmission energy.

The resulting structure defines a two-level communication hierarchy. Data are routed from each sensor to its CH through multi-hop paths over sensors and relays (Section 3.6), and then between CHs along the oriented MST to the sink.

### 3.9. Energy Consumption Calculation (Intra-Cluster and Inter-Cluster)

The energy consumption of the network was estimated for one data-collection round, in which each sensor generates one data packet. It was computed separately for the intra-cluster and inter-cluster communication components.

The first-order radio model of Heinzelman et al. [113], which is widely used to estimate the energy consumption of WSNs [137], was adopted, considering only the free-space propagation component. The energy required to transmit a packet of *k* bits over a distance *d* is:

E_TX_(k, d) = kE_elec_ + kE_amp_d^2^ (12)

The energy consumed in the reception of a packet is given by:

E_RX_(k) = kE_elec_ (13)

where

- E_elec_ = 50 × 10^−9^ J/bit is the energy consumed by the electronic circuits;
- E_amp_ = 100 × 10^−12^ J/bit/m^2^ is the energy required for signal amplification;
- k = 4000 bits is the packet size;
- d is the Euclidean distance between transmitter and receiver.

The adopted formulation corresponds to the simplified version of the radio model based on the free-space regime, in which the consumption associated with distance is proportional to *d^2^*.

In intra-cluster communication, energy consumption was calculated for each link of the multi-hop routes between the sensors and their respective CHs. Each packet is received and retransmitted by every intermediate node, sensor or relay, until it reaches the CH; relays do not generate packets. For a route consisting of H links, the energy required to deliver one packet is:

(14) $$E_{\mathrm{route}}=\sum_{h=1}^{H}\left[E_{\mathrm{TX}}\left(k,d_{h}\right)+E_{\mathrm{RX}}\left(k\right)\right]\;$$

The intra-cluster energy, *E_Intra_*, is the sum of *E_route_* over all sensors of all clusters. The energy consumed by relays, in transmission and reception, was also computed separately.

Inter-cluster energy. Under the aggregation assumption described in Section 3.8, each CH except the sink transmits one aggregated packet to its parent node along the MST. The inter-cluster energy is therefore:

(15) $$E_{\mathrm{Inter}}=\sum_{\left(i,j\right)\in \mathrm{MST}}\left[E_{\mathrm{TX}}\left(k,d_{\mathrm{ij}}\right)+E_{\mathrm{RX}}\left(k\right)\right]$$

where *d_ij_* is the length of MST link (*i*,*j*).

The total network energy consumption was then calculated as:

E_Total_ = E_Intra_ + E_Inter_ (16)

This formulation makes it possible to evaluate how the spatial distribution of sensors, the number of relay nodes, the formation of clusters, the selection of Cluster Heads and the inter-cluster communication structure affect energy consumption. Because the same model and parameters were applied to all scenarios, the results are primarily intended to compare configurations rather than to predict the absolute energy consumption of a deployed network.

Several simplifications should be noted. Only the free-space (*d*^2^) component was considered, although the multipath (*d*^4^) component of the extended model [138] would apply to the longer inter-cluster links of the idealized MST, so absolute values are likely underestimated. The energy cost of data aggregation at the CHs was not included. Finally, the first-order model is a generic abstraction and does not reproduce the specific energy profile of LoRa transceivers, which depends mainly on transmission power and time-on-air.

### 3.10. Stability and Uncertainty Analysis

A fixed seed makes a single run reproducible but does not show that its results are stable. Two sources of variability were therefore distinguished and quantified. Placement variability arises only in the random control, whose sensor positions depend on the sampling seed; the greedy heuristic is deterministic (ties between candidate positions with equal marginal gain were broken by the lowest column-major cell index), so the reference and alternative scenarios have no placement variability. Clustering variability arises in all scenarios, because the K-means solution depends on its initialization.

For the reference scenario, K-means (n_init = 10) was repeated with *N_c_* = 30 independent values of random_state for each k ∈ {28, 29, 30}, with the sensor positions held fixed. For the random control, *N_p_* = 100 independent placements were generated, and each was clustered with *N_c_* independent K-means seeds (k = 29). For every run, the coverage of Class 4–5 cells, the number of relays, the total energy per round and the energy per covered priority cell were recorded, and the results are reported as mean, standard deviation and 2.5–97.5 percentile interval. For the random control, the variance of each metric was decomposed, by the law of total variance, into a between-placement component (variance across placements of the per-placement mean) and a within-placement component (mean across placements of the variance across clustering seeds), which attribute the variation to placement and to clustering, respectively.

Differences among k = 28, 29 and 30 were interpreted as substantive only if the 95% interval of the difference in mean total energy across clustering seeds excluded zero and the ranking of the configurations was consistent across seeds; otherwise, they are reported as descriptive.

## 4. Results

### 4.1. Numerical Hazard Map of the Study Area

**Figure 4:**
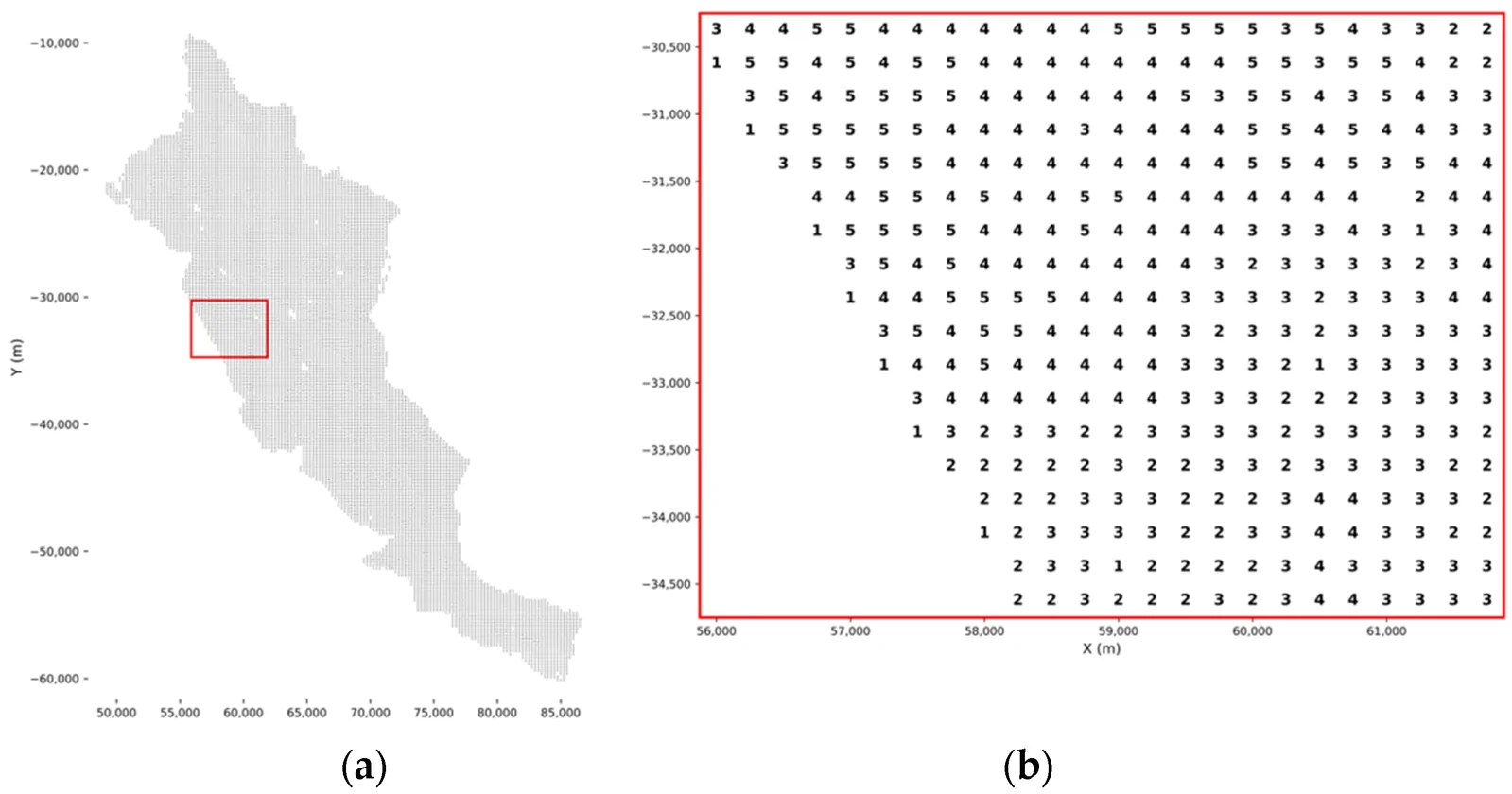
Numerical hazard map of the PNSSM discretized into a 250 × 250 m grid: (**a**) complete map, with the area of detail highlighted in red; (**b**) enlarged detail of the highlighted area, showing the hazard class (1–5) assigned to each cell.

The application of the methodology allowed the conversion of the Official Rural Fire Hazard Map of the ICNF into a matrix spatial representation of the Serra de São Mamede Natural Park. Figure 4a shows the reclassified hazard map, while Figure 4b shows its discretization into a regular grid composed of 250 × 250 m cells.

The area analyzed corresponds to 56,061.31 ha and was represented by a matrix of 150 × 204 cells. Each cell was associated with its respective Cartesian coordinates (X, Y) and the corresponding hazard level. The spatial distribution of the classes showed a heterogeneous pattern, with the areas of high and very high hazard (levels 4 and 5) concentrated in specific sectors of the PNSSM and interspersed with cells of lower hazard.

The comparison between Figure 3b and Figure 4a shows that the discretization preserves the main spatial patterns of the hazard map, including the main clusters of cells classified at levels 4 and 5. Of the total 30,600 cells in the matrix, 9043 (29.6%) had an assigned hazard class (1–5) and the remaining cells correspond to areas without classification, mostly outside the park boundary. Among the classified cells, 2995 (33.1%) belonged to hazard levels 4 and 5 and were classified as priority cells in the reference scenario.

These cells showed a non-uniform spatial distribution, forming clusters of higher concentration in different sectors of the park. The matrix representation therefore allowed for the quantitative identification of the priority spatial units used in the sensor positioning stage. The 2995 cells at levels 4 and 5 constituted the reference spatial set for the coverage assessment and for determining the monitoring positions in subsequent stages.

### 4.2. Identification of Priority Cells in the Three Scenarios

Based on the hazard matrix representation presented in Section 4.1, the cells classified at high (4) and very high (5) hazard levels were considered priorities. The spatial matrix comprises 30,600 cells, of which 9043 have an assigned hazard class and correspond to the park area (9043 cells × 6.25 ha ≈ 56,500 ha); the remaining cells lie mostly outside the park boundary. Of the classified cells, 2995 were identified as priority cells, which means that approximately one third (33.1%) of the PNSSM territory is classified as high or very high hazard. Class 3 (moderate hazard) accounts for a further 3312 cells (36.6%), and Classes 1–2 (very low and low hazard) for 2736 cells (30.3%). Accordingly, in the alternative scenario (classes 3–5), the priority set increased to 6307 cells (69.7% of the classified cells), whereas in the control scenario all 9043 classified cells were considered eligible for the random deployment.

As observed in Figure 4a, the priority cells showed a non-uniform spatial distribution, forming different clusters and concentration zones throughout the study area. This distribution indicates that the areas of greatest hazard, although accounting for approximately one third of the park’s classified cells, are concentrated in specific sectors rather than uniformly distributed across the territory.

The spatial distribution of priority cells defined the demand set for sensor placement in the reference and alternative scenarios. The reference scenario included 2995 cells classified as hazard Classes 4 and 5, whereas the alternative scenario expanded the demand set to 6307 cells by also including Class 3. Each priority cell was treated as a spatial unit requiring network coverage, ensuring that sensor placement reflected the distribution of areas with higher hazard levels. In contrast, the control scenario used all 9043 classified cells as the pool of eligible positions for random sensor deployment, without prioritizing locations according to hazard level. These scenario definitions provided the basis for the sensor placement analysis, the results of which are presented in Section 4.3.

### 4.3. Sensor Positioning in the Three Scenarios

In the reference scenario and considering a monitoring range of r = 1 (each sensor covering its own cell and the eight surrounding cells, Section 3.4.1), the optimization process was applied to the 2995 priority cells identified in the hazard matrix of 150 × 204 cells. The solution obtained selected 589 positions for sensor installation in the Serra de São Mamede Natural Park, covering a study area of 56,061.31 ha. The relationship between the number of priority cells and the number of selected positions results in an average ratio of approximately 5.1 priority cells per sensor (2995/589). This value represents the spatial relationship between the demand considered by the model and the number of sensors installed. However, it does not directly correspond to the effective number of cells monitored per sensor, since the coverage depends on the spatial configuration of the cells covered and the possible overlap between the monitoring areas.

**Figure 5:**
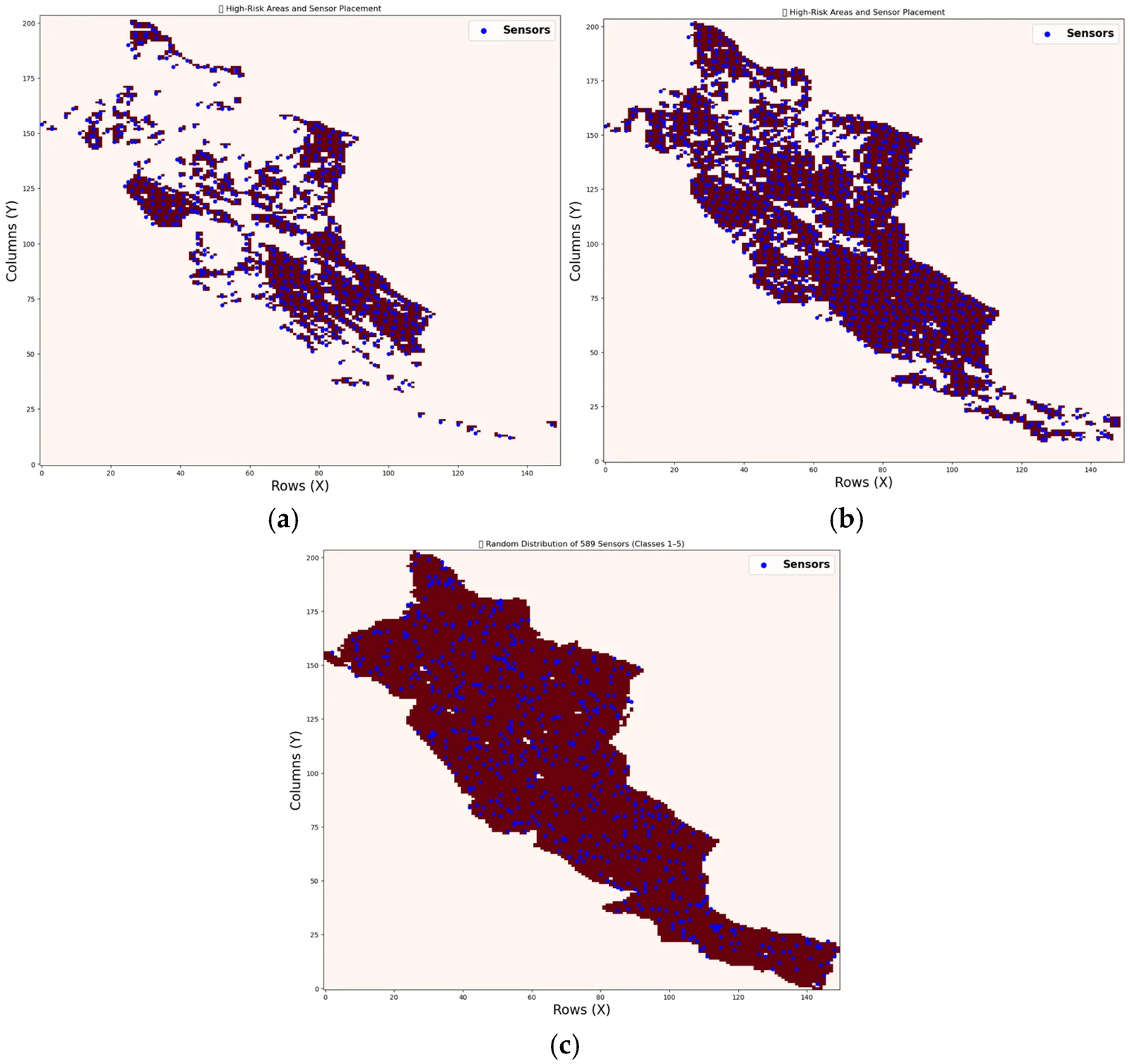
Sensor positions in the three scenarios: (**a**) reference scenario (Classes 4–5, 589 sensors); (**b**) alternative scenario (Classes 3–5, 1054 sensors); (**c**) control scenario (random deployment of 589 sensors over Classes 1–5).

The spatial distribution of the 589 selected positions is shown in Figure 5a. A higher concentration of sensors is observed in the regions where the cells classified at the highest hazard levels are located, while the lower priority areas show a lower density of positions. Consequently, the solution obtained presents a non-uniform spatial distribution of sensors, following the spatial configuration of the priority areas identified in the previous step.

The spatial analysis also shows that the selected positions are not evenly distributed among the 2995 priority cells. Some positions are found associated with areas of concentration of priority cells, allowing a single sensor to contribute to the coverage of several adjacent spatial units. This pattern results in a solution with 589 positions to represent a spatial demand composed of 2995 priority cells.

In the alternative scenario, the greedy heuristic selected 1054 positions to cover the 6307 priority cells of Classes 3–5, corresponding to an average of 6.0 priority cells per sensor (Figure 5b). Extending the priority set to Class 3 therefore increased the number of sensors by 78.9% for a demand set 110.6% larger and spread the sensors over a much larger part of the park, since Classes 3–5 occupy about 70% of the classified area.

In the control scenario, the same number of sensors as in the reference scenario (589) was randomly distributed over the 9043 classified cells (Figure 5c). Unlike in the optimized scenarios, these positions do not follow the distribution of hazard, so sensors were placed in cells of all hazard classes, approximately in proportion to their extent.

The sensor configurations obtained in the three scenarios, with 589, 1054 and 589 sensors, respectively, constitute the input for the subsequent steps of cluster formation, Cluster Head selection and definition of communication routes.

### 4.4. Coverage Assessment and Spatial Validation

**Figure 6:**
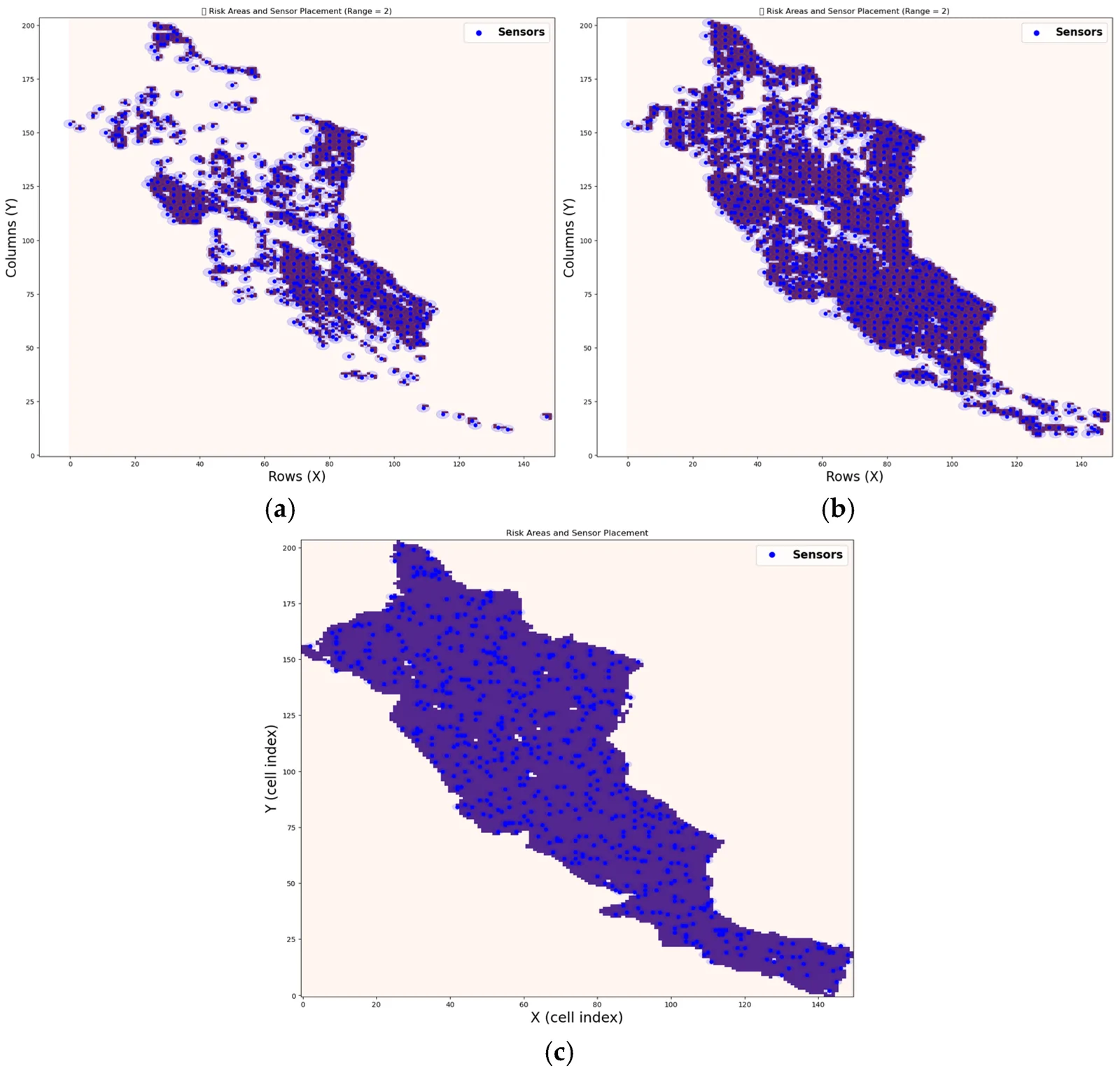
Sensor positions and coverage (r = 1) in the three scenarios: (**a**) reference scenario, with the priority cells of Classes 4–5 and 589 sensors; (**b**) alternative scenario, with the priority cells of Classes 3–5 and 1054 sensors; (**c**) control scenario, with the eligible cells of Classes 1–5 and 589 randomly placed sensors. Dark red: priority cells (**a**,**b**) or eligible cells (**c**); blue dots: sensors; light blue halos: coverage area of each sensor.

Coverage was evaluated with the criterion defined in Equation (1), in which each sensor covers its own cell and the eight surrounding cells (r = 1), corresponding to center-to-center distances of 250 m for orthogonal neighbors and approximately 354 m for diagonal neighbors. Figure 6 represents, for each scenario, the cells considered in the placement step, the sensor positions, and the coverage area of each sensor. These cells are the Class 4–5 priority cells in the reference scenario (Figure 6a), the Class 3–5 priority cells in the alternative scenario (Figure 6b), and all classified cells (Classes 1–5), eligible for the random deployment, in the control scenario (Figure 6c).

The application of the greedy MCP resulted in the selection of 589 sensor positions, ensuring coverage of the 2995 priority cells identified in Section 4.2. Thus, considering the coverage criterion defined in the problem formulation, 100% coverage of the priority cells was achieved. On average, each selected sensor contributes to the coverage of approximately 5.1 priority cells. In the alternative scenario, the 1054 sensors likewise covered all 6307 priority cells of Classes 3–5 and, consequently, all 2995 cells of Classes 4–5.

In Figure 6a,b, the sensors follow the shape of the priority areas. They are densely packed where the priority cells form continuous patches, and more widely spaced where these cells are scattered, particularly in the peripheral sectors of the park. In the alternative scenario, the inclusion of Class 3 extends the network over most of the park, so that the sensor pattern in Figure 6b approaches the outline of the classified area.

In the control scenario (Figure 6c), the sensors are spread over the whole park, irrespective of the hazard level, and coverage is not guaranteed by construction. Because this map represents all eligible cells, it does not distinguish the priority cells left uncovered. Their coverage was therefore quantified with the same criterion (Equation (1)). Only 45.9% of the Class 4–5 cells, 45.2% of the Class 3–5 cells and 44.5% of all classified cells were covered in the draw with seed = 42 (Class 4–5 coverage over 100 random placements: 45.3 ± 1.5%, 2.5–97.5% interval 42.6–48.0%; Section 4.10.1). With the same number of sensing nodes, the reference scenario therefore covered about 2.2 times as many high- and very-high-hazard cells as the random deployment (2995 versus approximately 1375 cells). This difference quantifies the combined effect of hazard prioritization and coverage optimization relative to random placement, for an equal number of sensing nodes and it is independent of the network organization steps analyzed in the following sections. The separate contributions of the two components, under a common feasible domain, are examined in Section 4.10.2.

### 4.5. Cluster Formation and Cluster Head Selection

**Figure 7:**
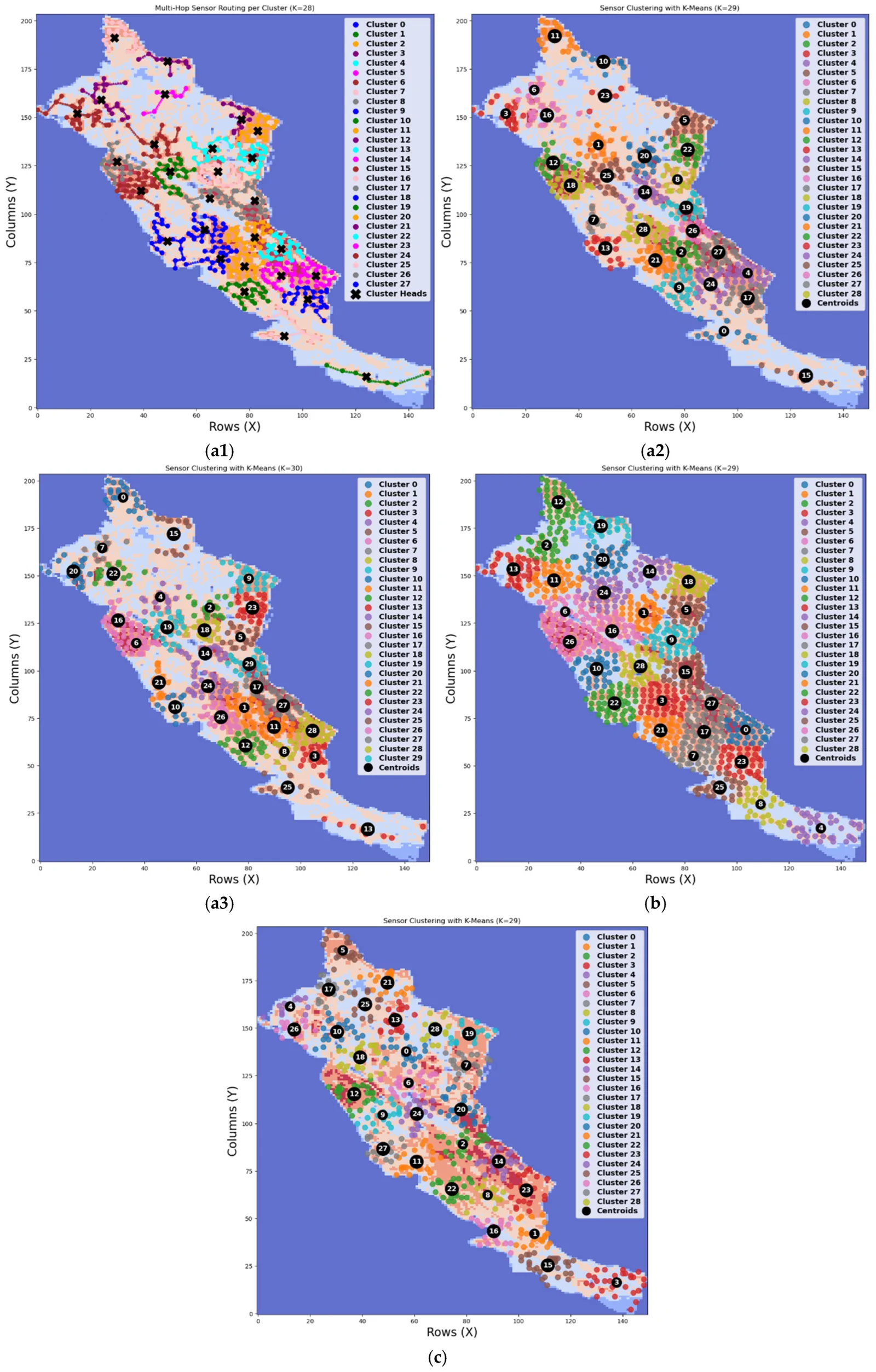
K-means cluster configurations: (**a1**–**a3**) reference scenario with 28, 29 and 30 clusters, respectively; (**b**) alternative scenario (Classes 3–5, k = 29); (**c**) control scenario (random deployment, k = 29).

After sensor positioning, the hierarchical organization of the network was carried out using the K-means algorithm. In the reference scenario, three configurations with 28, 29 and 30 clusters were evaluated to assess the sensitivity of the network to k, whereas the alternative and control scenarios were organized with k = 29. Figure 7 shows the spatial distribution of the clusters obtained for the three configurations of the reference scenario (Figure 7(a1–a3)) and for the alternative and control scenarios (Figure 7b,c). In the reference scenario, the clusters follow the patches of high and very high hazard, whereas in the alternative and control scenarios they extend over most of the park, reflecting the wider spatial distribution of the sensors. In all cases, the groupings are consistent with the location of the sensors.

In each configuration and scenario, the selected CH corresponded to the physical sensor closest to the geometric centroid of the respective cluster. Thus, all clusters were associated with an effectively existing node in the network, without the introduction of virtual positions.

The distribution of the number of sensors per cluster showed some heterogeneity among the three configurations. For 28 clusters, each grouping had, on average, 21.0 ± 5.6 sensors, with values between 8 and 30 sensors. For 29 clusters, the average was 20.3 ± 6.6 sensors, ranging from 7 to 32 sensors. In the configuration with 30 clusters, an average of 19.6 ± 5.8 sensors per cluster was observed, with values between 8 and 28 sensors provided in Table 1. These results show a progressive reduction in the average size of the clusters as the number of groupings increases.

The average distance between the sensors and their respective CHs also decreased with the increase in the number of clusters. The average value went from 1494 m to 1440 m and 1425 m in the configurations with 28, 29, and 30 clusters, respectively. Between the configurations with 28 and 30 clusters, there was a reduction of 69 m, corresponding to approximately 4.6% of the average distance observed in the reference configuration. The reduction was accompanied by a decrease in the average number of sensors per cluster, from 21.0 to 19.6.

In the configuration with 29 clusters, for example, the average distance of 1440 m corresponds to approximately 5.8 grid cells. This distance is almost six times the communication range assumed between sensors (250 m), which indicates that direct communication between each sensor and its CH is not compatible with the adopted range and that intra-cluster communication requires multi-hop routing. Overall, the results show that increasing the number of clusters from 28 to 30 produces a moderate reduction in both the average cluster size and the average distance between sensors and their CHs. The configuration with 30 clusters presented the lowest values for both metrics, with 19.6 sensors per cluster and an average sensor-to-CH distance of 1425 m. However, the difference between the configurations is relatively small, so the selection of the final configuration should also consider the costs associated with inter-cluster communications and routing, analyzed in the following sections.

In the alternative scenario, the larger number of sensors organized into the same 29 clusters resulted in clusters with 36.3 ± 4.3 sensors (27–48), almost twice the size targeted by the criterion adopted in Section 3.5, and in a longer average sensor-CH distance (1649 ± 680 m). In the control scenario, the clusters had 20.3 ± 4.1 sensors (12–30) and were therefore more uniform in size than in the reference scenario, but the average sensor-CH distance was also longer (1585 ± 750 m, compared with 1440 m in the reference scenario with k = 29), because the random sensors are spread over the whole park rather than concentrated in the high-hazard sectors.

### 4.6. Intra-Cluster Connectivity, Relay Nodes and Multi-Hop Routing

**Figure 8:**
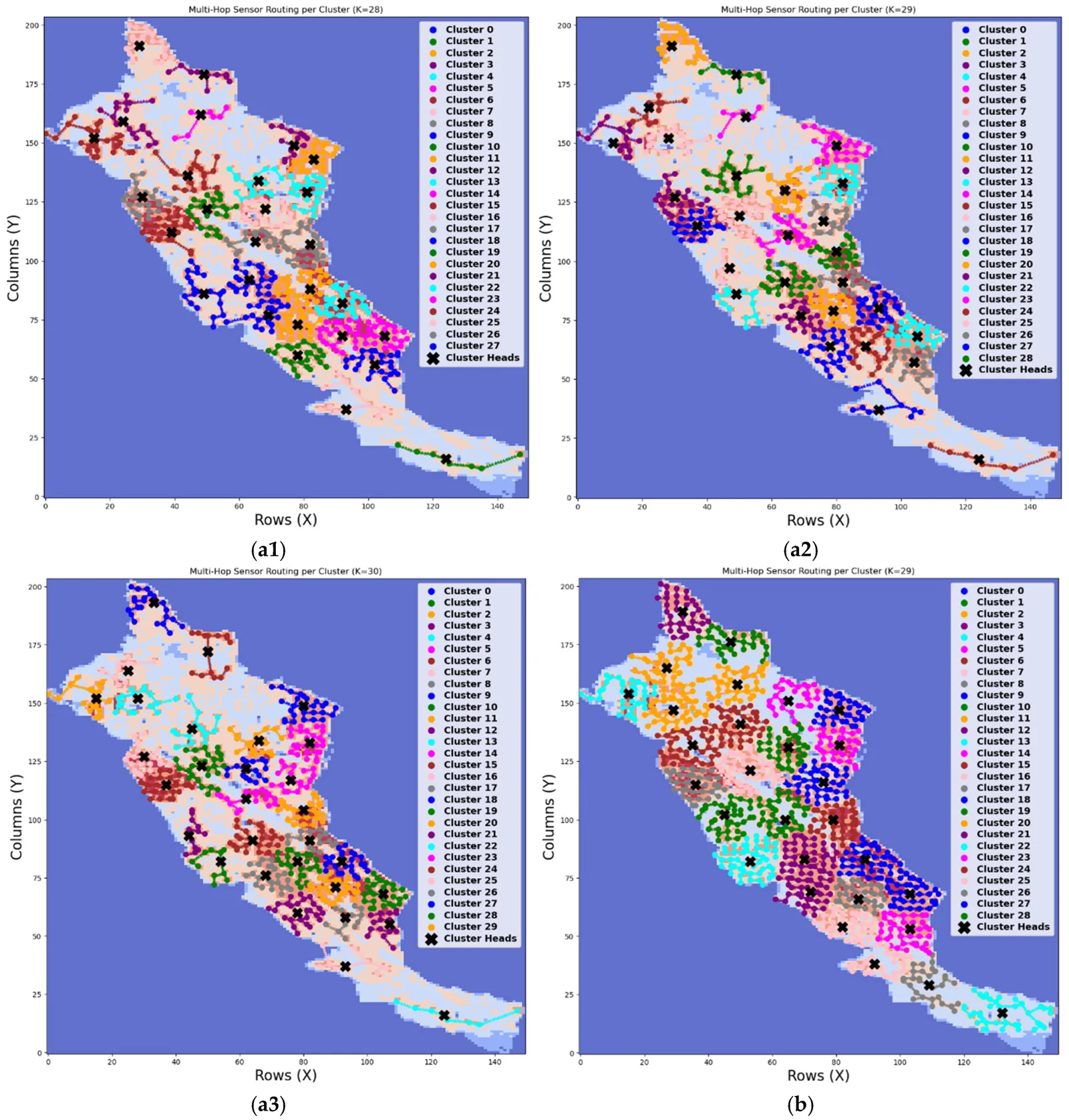

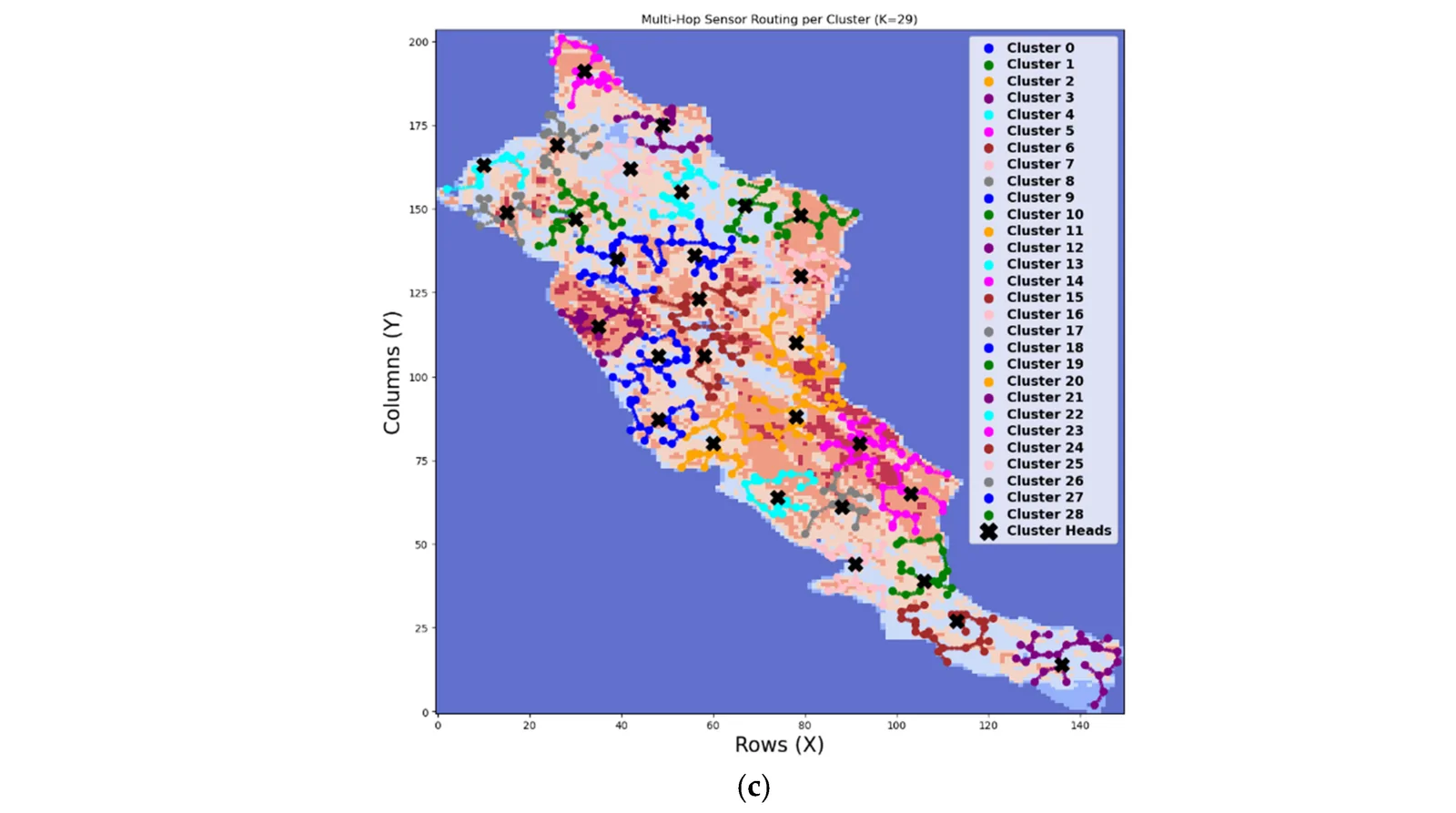
Multi-hop routing structure, including relay nodes: (**a1**–**a3**) reference scenario with 28, 29 and 30 clusters, respectively; (**b**) alternative scenario (k = 29); (**c**) control scenario (k = 29).

The routing structures obtained for the three configurations of the reference scenario are shown in Figure 8(a1–a3), and those of the alternative and control scenarios in Figure 8b,c. In all configurations and scenarios, the established paths follow the spatial distribution of the sensors and progressively connect the nodes to their respective Cluster Heads (CHs) through intermediate sensors and relay nodes.

The positioning step spaced the sensors to maximize coverage, so most of them had no neighboring sensor within the 250 m communication range: the median distance from each sensor to its nearest neighbor was 559 m, and only 14.3% of the sensors had another sensor within 250 m. Without relay nodes, about 95% of the sensors (557 of 589 for k = 30) would have no multi-hop path to their CH. The relay placement described in Section 3.6 removed this limitation, requiring 1174, 1159 and 1169 relays for 28, 29 and 30 clusters, respectively, that is, about two relays per sensor. With relays, every sensor obtained a route to its CH in all three configurations, and no link exceeded 250 m.

The routes were relatively long. The mean number of hops per sensor was 12.2, 11.4 and 11.7 for 28, 29 and 30 clusters, with maxima of 45, 34 and 48 hops, respectively. Relays accounted for approximately two thirds of the intra-cluster energy (66.2%, 65.3% and 66.7%), which shows that the connectivity cost induced by the 250 m range is a major component of the intra-cluster energy budget.

Table 2 summarizes the intra-cluster connectivity of the three scenarios. In the alternative scenario, 1751 relays were required, corresponding to 1.66 relays per sensor, fewer per sensor than in the reference scenario (1.97 for k = 29) because the denser deployment shortens the gaps between sensors. Nevertheless, the larger clusters produced longer routes, with 18.0 hops per sensor on average and a maximum of 71. In the control scenario, 1208 relays were required (2.05 per sensor), the highest ratio among the three scenarios, with 12.7 hops per sensor on average (maximum 51). In all scenarios, relays accounted for 63–67% of the intra-cluster energy, and every sensor obtained a route to its CH with links no longer than 250 m.

The spatial distribution of the links showed differences between configurations and scenarios. In regions with a higher concentration of sensors, routing structures with a higher density of connections were observed, while in clusters located in peripheral regions or with a lower density of nodes, the paths showed a more elongated configuration. In the alternative scenario, the larger clusters produced longer and more branched routes, whereas in the control scenario the routes cover the whole park more uniformly. These differences reflect the spatial distribution of the sensors resulting from the positioning stage and the subsequent formation of the clusters.

The use of multi-hop routing over sensors and relays therefore allowed establishing paths between sensors and their respective CHs even when the direct distance between them exceeded the communication range by several times, ensuring the continuity of intra-cluster paths at the cost of a large number of additional nodes.

### 4.7. Inter-Cluster Structure

Connectivity between the Cluster Heads was subsequently established through a Minimum Spanning Tree (MST). The number of edges in the MST was 27, 28, and 29 for the configurations with 28, 29, and 30 clusters, respectively, corresponding to the minimum number of connections needed to connect all the CHs. The average length of the MST links was 4098 ± 1451 m, 4158 ± 1409 m and 3977 ± 1412 m, respectively, and the total length of the tree was 110.65 km, 116.44 km and 115.34 km (Table 3). In all configurations, the longest link measured 9361 m and connected an isolated cluster in the south-western sector of the park.

The configuration with 29 clusters presented the greatest average and total length of the MST links (4158 m and 116.44 km), while the configuration with 30 clusters presented the lowest average length (3977 m). The inter-cluster link lengths therefore varied as a function of the spatial distribution of the CHs and did not show a monotonic trend with the number of clusters. The longest links, close to 9.4 km, exceed by far the 250 m sensor-to-sensor range and are not substantiated as feasible radio links in the study area; the unconstrained MST is therefore interpreted only as an idealized connected graph (Section 3.8). The topology recomputed under the 250 m link constraint is presented in Section 4.10.3.

In the alternative and control scenarios (k = 29), the MST had a total length similar to that of the reference scenario (117.89 km and 114.64 km), but a much more regular structure, with standard deviations of the link length of 570 m and 809 m, compared with 1409 m in the reference scenario. Their longest links (6486 m and 6605 m) were also shorter than in the reference scenario (9361 m), because the sensors of these scenarios occupy a larger part of the park and leave fewer isolated groups of CHs.

Taking together, the results show that, with the addition of relay nodes, all configurations and scenarios produced complete intra-cluster routing structures, without sensors disconnected from their respective CHs, and inter-cluster structures connected through the MST.

### 4.8. Selected Sink Node

**Figure 9:**
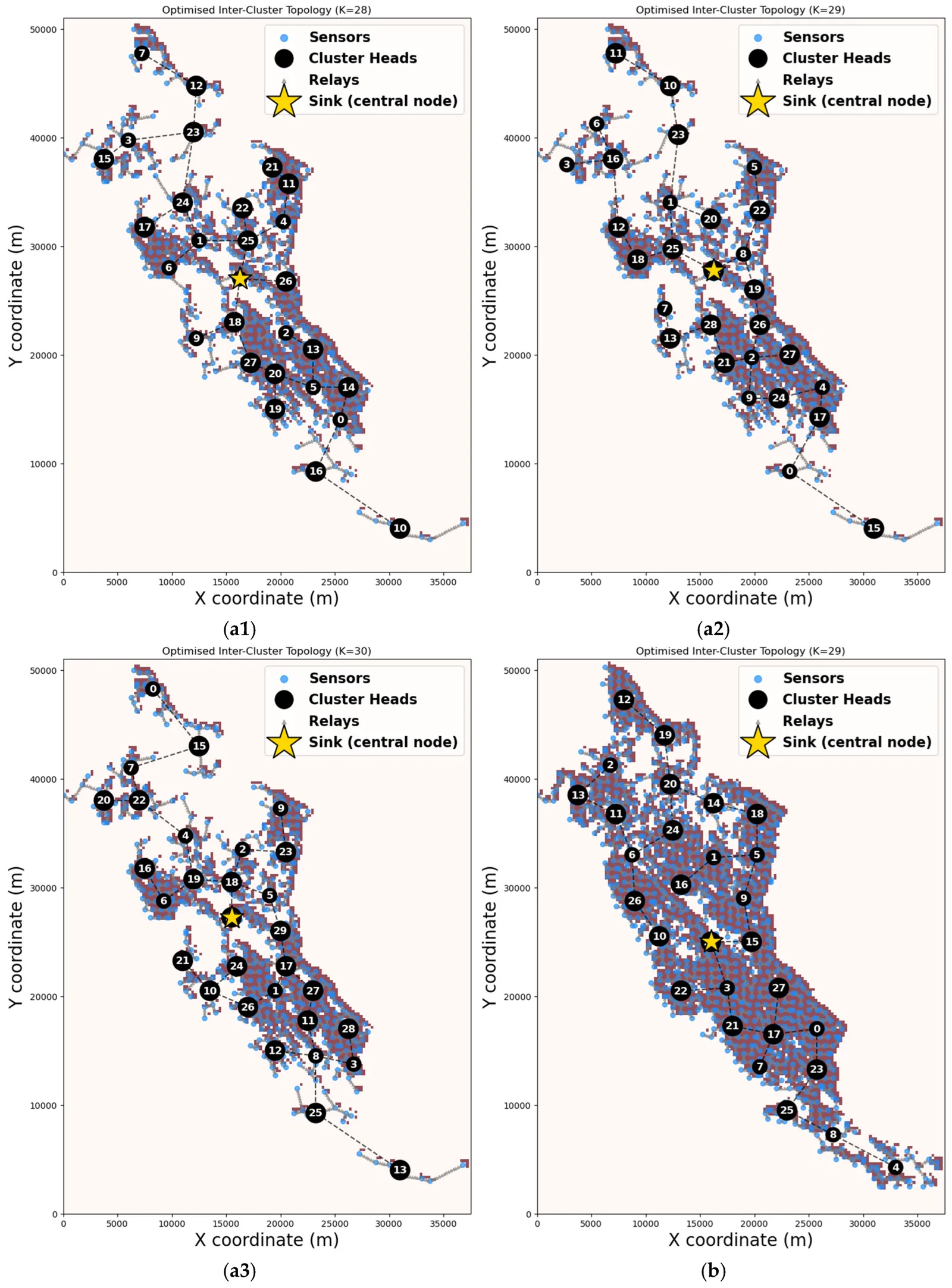

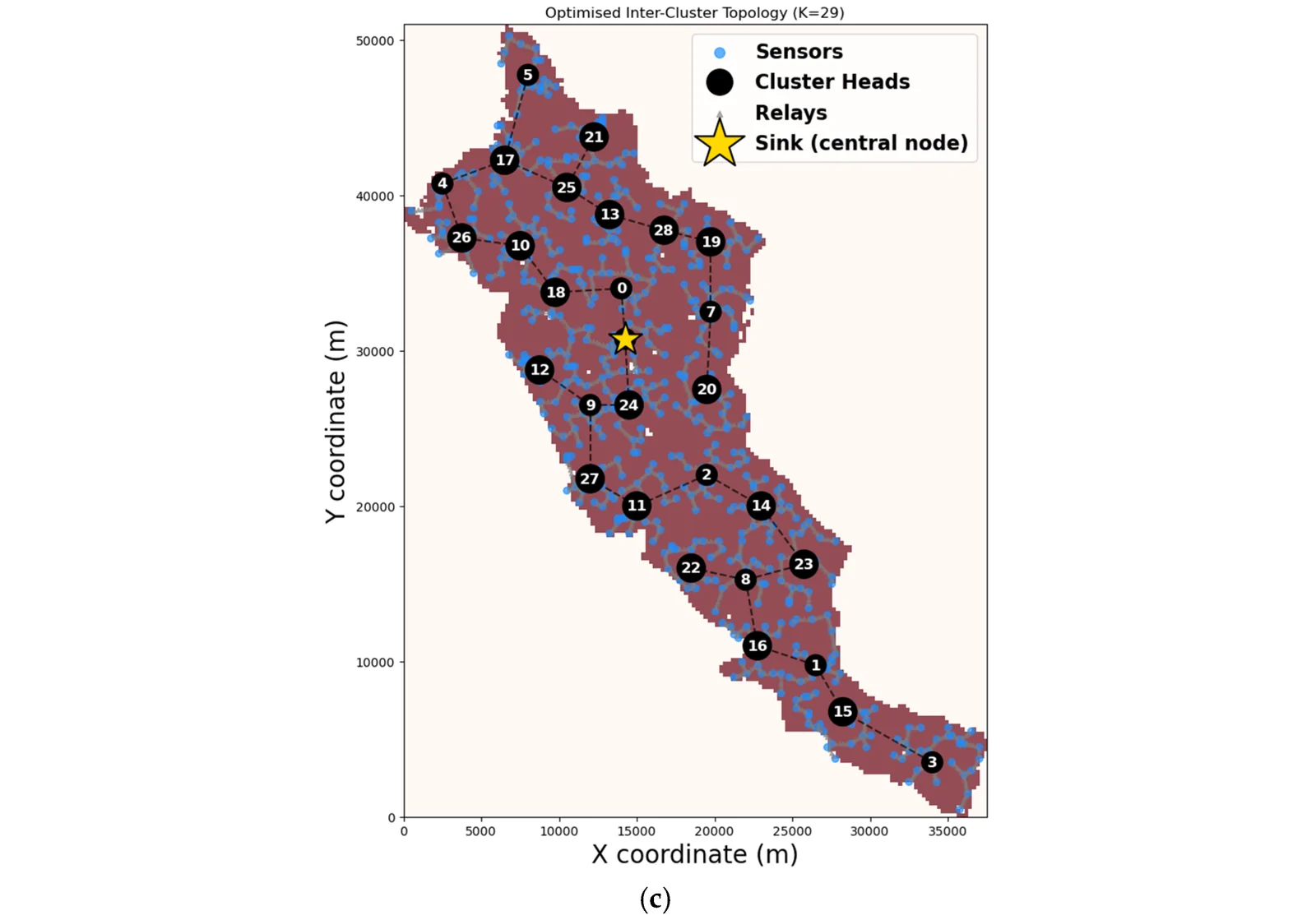
Inter-cluster MST and sink node: (**a1**–**a3**) reference scenario with 28, 29 and 30 clusters, respectively; (**b**) alternative scenario (k = 29); (**c**) control scenario (k = 29). The star marks the sink. Numbers identify the clusters (Cluster Head indices).

In the configurations with 29 and 30 clusters, the algorithm selected the CH of Cluster 14 as the sink, located at positions (65, 111) and (62, 109) of the spatial matrix, respectively. For the configuration with 28 clusters, the CH of Cluster 8 was selected, located at position (65, 108). Along the MST, the mean number of hops between each CH and the sink was 3.9, 4.6 and 5.2 for 28, 29 and 30 clusters, with maxima of 8, 10 and 9 hops, respectively. Figure 9 shows the location of the sink in the three configurations. In the alternative and control scenarios, the sink was the CH of Cluster 28, at position (64, 100), and the CH of Cluster 6, at position (57, 123), respectively, with 5.5 and 6.5 hops on average between each CH and the sink (maxima of 11 and 12; Table 3).

In all configurations, the selected sink corresponded to an existing CH in the network and was located close to the central region of the spatial distribution of the CHs. Despite the variation in the number of clusters between 28, 29, and 30, the spatial location of the sinks remained concentrated in a similar central region of the network.

The comparison between the three configurations therefore shows high spatial stability in the selection of the sink. The change in the number of clusters from 28 to 30 did not produce a substantial change in the spatial region where the collection point was identified. In the 29 and 30 cluster configurations, selecting the same CH as a sink reinforces this stability.

The location of the sink in relation to the remaining CHs determines the depth of the inter-cluster tree, that is, the number of hops that data from the most distant CHs must traverse. Under the aggregation assumption adopted (one aggregated packet per MST link), the inter-cluster energy does not depend on which CH is selected as the sink, since the MST and the number of transmissions per link are the same. The sink location therefore influences mainly the number of hops and, consequently, latency, rather than the estimated energy consumption.

### 4.9. Energy Consumption of the Evaluated Configurations

The energy efficiency of the proposed architecture was evaluated based on the first-order radio model of Heinzelman et al. [113], jointly considering the consumption associated with intra-cluster and inter-cluster communications during a complete monitoring round. The total energy consumption was calculated from the energy spent on transmissions and receptions necessary for routing data between the sensors, the relay nodes, the respective Cluster Heads (CHs) and the sink.

The energy results of the three scenarios are presented in Table 4. In the reference scenario (single clustering run, random_state = 42), the configuration with 30 clusters showed the lowest total energy consumption, with 343.20 J per round, followed by the configuration with 28 clusters, with 347.36 J. The configuration with 29 clusters showed the highest consumption, with 349.59 J. The inter-cluster component accounted for 58.8%, 61.8% and 60.2% of the total consumption, respectively, reflecting the long CH-to-CH links of the idealized MST (Section 3.8).

Compared with the configuration with 28 clusters, the configuration with 29 clusters increased energy consumption by 2.23 J (0.6%), while the configuration with 30 clusters reduced it by 4.16 J (1.2%). Between the configurations with 29 and 30 clusters, the reduction was 6.40 J (1.8%).

The results therefore do not show a monotonic trend with the number of clusters. The intra-cluster component decreased from 143.20 J to 133.66 J between 28 and 29 clusters, in parallel with the reduction in the average number of sensors per cluster (from 21.0 to 20.3) and in the average sensor-CH distance (from 1494 m to 1440 m) and increased slightly for 30 clusters (136.56 J).

The inter-cluster component, in contrast, increased from 204.16 J to 215.94 J between 28 and 29 clusters and decreased to 206.64 J for 30 clusters, following the total length of the MST (110.65 km, 116.44 km and 115.34 km). Because the energy of each link grows with the square of its length, a few long links weighs heavily on this component. The variation in energy consumption between configurations therefore results from the interaction between intra- and inter-cluster communication structures and cannot be attributed exclusively to the number of clusters.

The three configurations maintained coverage of the 2995 priority cells identified in Section 4.2 and presented intra-cluster and inter-cluster connectivity, as shown in Section 4.6 and Section 4.7. In this run, the configuration with 30 clusters presented the lowest energy consumption among the evaluated values of k, with 343.20 J per monitoring round, corresponding to a 1.2% reduction compared to the configuration with 28 clusters and a 1.8% reduction compared to the configuration with 29 clusters. These differences are, however, small and are treated as descriptive: across independent K-means seeds (Section 4.10.1), the standard deviation of the total energy within each k was about 7 J, comparable to or larger than the differences between configurations of this run. In means over 30 seeds, k = 28 consumed about 2% more than k = 29 and k = 30, whereas k = 29 and k = 30 did not differ (1.1 J; 95% CI −2.4 to 4.7 J), so the higher consumption of k = 29 in this run is not a stable result. Within this range, the choice of k therefore has a limited effect on the estimated consumption, and no configuration is identified as optimal.

With the cluster count fixed at k = 29, the alternative scenario consumed 596.27 J per round, 70.6% more than the reference scenario with the same k, mainly because of the intra-cluster component (394.06 J versus 133.66 J), driven by the larger number of sensors and relays (1751) and by longer routes (18.0 hops per sensor on average, with a maximum of 71). Its energy per sensor (566 mJ) was, however, slightly lower than in the reference scenario (594 mJ), because a denser deployment shortens the gaps that relays must bridge (1.66 relays per sensor, compared with 1.97 in the reference scenario). Its inter-cluster component (202.21 J) was also lower, with a longest MST link of 6486 m. This increase is conditional on the fixed cluster count, which raised the average cluster size from 20.3 to 36.3 sensors and the average route length from 11.4 to 18.0 hops; it is the result at k = 29 and not a general cost of including Class 3. With k = 53, which restores approximately 20 sensors per cluster, the increase was 21.0%, compared with 63.4% at k = 29 under the same multi-seed procedure (Section 4.10.4).

In the draw with seed = 42, the random deployment consumed 339.40 J per round, 2.9% less than the reference scenario (349.59 J). This difference results from the inter-cluster component (195.09 J versus 215.94 J): sensors spread over the whole classified area produced a more regular arrangement of CHs, with a longest link of the idealized MST of 6605 m, compared with 9361 m in the reference scenario. The intra-cluster component was slightly higher in the random scenario (144.31 J versus 133.66 J), which required 1208 relays. Energy consumption alone therefore does not favour the hazard-oriented deployment. However, when energy is related to the monitoring outcome, the reference scenario consumed approximately 117 mJ per covered high- or very-high-hazard cell, compared with approximately 247 mJ in the random deployment, that is, about half the energy per covered priority cell. Over all random placements and clustering seeds, the energy per covered priority cell of the random control was 250.8 ± 12.2 mJ, compared with 113.5 ± 2.4 mJ for the reference scenario, and the mean total energy of the two scenarios was practically identical (339.8 ± 11.9 J and 339.9 ± 7.2 J; Section 4.10.1).

These results indicate that the main benefit of the combined procedure (hazard prioritization and coverage optimization), relative to random placement, lies in where the monitoring effort is placed, rather than in a reduction in the network’s energy consumption. For an equal number of sensing nodes (but not an equal total number of deployed nodes) and a similar energy budget, it more than doubles the coverage of the areas of greatest fire hazard.

### 4.10. Stability and Supplementary Analyses

This section reports the analyses introduced in Section 3.4.4, Section 3.8, Section 3.5 and Section 3.10 to test the stability of the results, the attribution of the coverage gain, the feasibility of the inter-cluster links and the dependence of the alternative scenario on the cluster count.

#### 4.10.1. Stability Across Placement and Clustering Seeds

Table 5 summarizes the distribution of the main metrics over *N_c_* = 30 K-means seeds for the reference scenario and over *N_p_* = 100 placements × *N_c_* = 30 K-means seeds (3000 runs) for the random control. The supplementary analyses were run with a re-implementation of the pipeline of Section 3.3, Section 3.4, Section 3.5, Section 3.6, Section 3.7, Section 3.8 and Section 3.9 that reproduces the 589 sensors of the reference scenario exactly (1057 instead of 1054 sensors in the alternative scenario, owing to tie-breaking). Because the K-means solution also depends on the order of the input points and on the software version, single runs are not identical to those of Table 1, Table 2, Table 3 and Table 4, but the values of Table 1, Table 2, Table 3 and Table 4 lie within the distributions reported here (for example, 349.59 J for k = 29 lies at the 83rd percentile of the reference distribution, and 339.40 J for the random draw at the 49th percentile of the random-control distribution).

Coverage of the reference scenario is 100% by construction and does not depend on clustering. For the random control, 51.5% of the variance of the total energy was attributable to placement and 48.5% to clustering; for the relay count the shares were 87.5% and 12.5%, and for the energy per covered priority cell 74.7% and 25.3%, whereas the variance of coverage is due to placement only. Within each k, the total energy of the reference scenario had a standard deviation of about 7 J across clustering seeds, comparable to or larger than the 2.2–6.4 J differences between k = 28, 29 and 30 in the single run of Section 4.9. In means over the 30 seeds, k = 28 consumed 6.5 J (1.9%) more than k = 29 (95% CI 2.9–10.1 J) and 7.6 J (2.2%) more than k = 30 (95% CI 4.1–11.1 J), whereas k = 29 and k = 30 did not differ (1.1 J, 95% CI −2.4 to 4.7 J); k = 29 and k = 30 each gave the lowest energy in 47% of the seeds, and k = 28 in 7%. The ranking of the single run (k = 29 highest) is therefore not stable, and the differences among cluster counts are treated as descriptive. Across placements, the coverage gain of the reference scenario over random placement was 54.7 percentage points (2.5–97.5% interval 52.0–57.4), and its energy per covered priority cell was 2.2 times lower (113.5 versus 250.8 mJ), while the mean total energy of the two scenarios was practically identical (339.9 versus 339.8 J).

#### 4.10.2. Results Under a Common Feasible Domain and for the Hazard-Blind Control

In the unrestricted reference solution, only 3 of the 589 sensors were located in unclassified cells. With the candidate positions restricted to the 9043 classified cells (scenario i-c), the greedy heuristic again required 589 sensors to cover all 2995 priority cells, and the network metrics were practically unchanged (Table 6). The difference in feasible domain between the optimized scenarios and the random control therefore has no material effect on the results. The hazard-blind, coverage-optimized control (scenario iv), with the same 589 sensing nodes and the same feasible positions as the random control, covered 61.1 ± 1.1% of the Class 4–5 cells (59.5–63.3% over the 20 tie-breaking seeds). Relative to random placement (45.3%), coverage optimization alone therefore accounts for 15.8 of the 54.7 percentage points of the coverage gain, and hazard prioritization for the remaining 38.9 percentage points (about 71%). Only this second component can be attributed to the use of the hazard map. Because it spreads sensors evenly over the whole park, the hazard-blind control also required more relays (1626 ± 14) and more energy per round (363.1 ± 10.3 J) than the other scenarios.

#### 4.10.3. Link-Constrained Inter-Cluster Topology

When the 250 m constraint is also applied to the inter-cluster layer, every CH-to-CH link satisfies the same range assumption as the intra-cluster links. The inter-cluster layer then required 447 ± 8 additional relays in the reference scenario with k = 29 (Table 7), raising the total number of deployed nodes to about 2200, and the inter-cluster energy decreased from 207.9 ± 6.1 J to 11.3 ± 0.2 J per round. This decrease is expected under the adopted model: the free-space amplifier term grows with d^2^, so replacing a link of length d by n hops of length d/n divides its amplifier energy by n, whereas each additional hop adds only the electronics cost of one transmission and one reception. The energy values obtained with the idealized MST therefore overstate the inter-cluster energy of a feasible network while understating the number of nodes it requires. Under the link constraint, the intra-cluster component accounts for about 92% of the total energy, and the ranking of the scenarios changes: with the idealized MST, the reference and random scenarios had practically the same mean total energy, whereas with the link constraint the reference scenario consumed 143.3 ± 5.2 J and the random control 151.8 ± 7.3 J per round (5.6% more; the random control consumed less than the reference mean in only 11% of the runs). Statements about total energy and deployment size should be based on this variant, whereas the idealized variant only shows that the CHs can be connected.

#### 4.10.4. Alternative Scenario with k = 53

The 70.6% energy increase of the alternative scenario reported in Section 4.9 was obtained with the cluster count fixed at k = 29, which produced clusters of 36.3 sensors on average. Under the multi-seed procedure, the increase at k = 29 was 63.4% (555.3 versus 339.9 J). With k = 53, which follows the criterion of approximately 20 sensors per cluster used for the reference scenario, the clusters had 19.9 sensors on average (SD within a run 4.1), the average route length fell from 16.5 to 10.7 hops, and the total energy was 411.3 ± 9.7 J per round, 21.0% more than the reference scenario with k = 29 (Table 8). Under the link-constrained variant, the corresponding increases were 152% (k = 29) and 64% (k = 53), because the intra-cluster component then dominates. The cost of extending the hazard threshold to Class 3 therefore depends strongly on the cluster count retained, and the value at fixed k = 29 should not be generalized. This is a focused check of the alternative scenario under the criterion of Section 3.5, not a search for the optimal cluster count.

## 5. Discussion

The results obtained demonstrate that the proposed methodology allows the integration, in a single methodological sequence, of spatial information on fire hazard, sensor positioning, hierarchical network organization, and communication structure. The main contribution of the study does not, therefore, lie in the isolated use of MCP, K-means, relay-assisted minimum-energy routing, or MST, techniques individually consolidated in the field of Wireless Sensor Networks, but in their integration into an architecture whose spatial configuration is initially guided by the distribution of fire hazard. This integration establishes a relationship between the environmental context of the territory and the spatial and topological characteristics of the network.

The use of official hazard mapping from the ICNF as input information for the positioning process constitutes one of the main differentiating characteristics of the proposed approach. The use of Geographic Information Systems (GIS) and hazard mapping has been widely recognized as a tool to support prevention and spatial prioritization of resources in forest fire risk management [9,139,140,141]. In parallel, studies on WSN have sought to optimize the deployment of sensors based on criteria such as coverage, connectivity, distance between nodes and energy consumption [24,26,92,93,94,95,96].

In the present study, these two dimensions are articulated through the direct use of hazard mapping in the positioning problem. The spatial classification of the territory, therefore, ceases to be merely a characterization of the study area and begins to play an operational role in defining priority monitoring locations. The identification of the 2995 cells classified as high and very high hazard allowed us to restrict the search space of the optimization process, directing network resources to the areas considered most relevant in the context of fire detection.

This strategy differs from approaches in which sensors are distributed uniformly, randomly, or according to predominantly geometric criteria. Although these strategies may produce networks with favorable coverage or connectivity properties, they do not necessarily distinguish regions with different levels of environmental priority. The proposed methodology therefore introduces an additional spatial dimension to the WSN design process, causing the distribution of monitoring resources to be conditioned by the spatial heterogeneity of the hazard.

The result of the full coverage of priority cells must, however, be interpreted according to the problem formulation. Achieving 100% coverage of the 2995 priority cells is a direct result of the stopping criterion of the greedy MCP algorithm, which continues as long as there are uncovered priority cells. Therefore, this value represents a property of the optimization solution and should not be interpreted as an independent demonstration of detection performance. This distinction is particularly relevant when compared with studies that present coverage percentages obtained under different formulations and experimental conditions. Haque and Soliman [21], for example, reported coverage greater than 96% in an adaptive hierarchical architecture, but their respective coverage criteria are not necessarily equivalent to those used in the present study. The control scenario provides an independent reference for this value: with the same 589 sensing nodes, random deployments covered only 45.3 ± 1.5% of the priority cells over 100 placements (45.9% in the draw with seed = 42).

Literature also shows that heuristic and bio-inspired methods can improve different properties of WSNs. Coverage improvements through an adaptive variant of PSO were reported in [24], while simultaneous gains in coverage, connectivity and robustness with an EPSO-based approach were shown in [142]. Other works also report benefits of optimization techniques for energy efficiency and topological organization of WSNs [98,143]. The main distinction of the approach developed in this study is, however, the fact that the optimization does not consider the deployment space as spatially undifferentiated, but the spatial priority is defined beforehand from an official source of information on fire hazard.

In this way, the methodological contribution consists not only in obtaining high coverage, but in establishing where this coverage should be prioritized. This distinction is particularly relevant in extensive areas, where the installation of sensors throughout the territory may be technically or economically unfeasible.

The influence of hazard mapping does not end with the selection of sensor positions. Since the distribution of sensors results from a process driven by priority cells, all subsequent network organization steps are performed on a non-uniform spatial configuration. Clustering through K-means, selection of CHs, and definition of routing structures are, consequently, conditioned by the geometry resulting from the initial positioning step.

This chaining constitutes a relevant characteristic of the proposed architecture. In conventional approaches, clustering is often applied to a previously defined sensor distribution based on geometric or random criteria. In the present study, on the contrary, the formation of clusters occurs on a network whose spatial distribution already incorporates information related to hazard. Thus, the heterogeneity observed in the size of the clusters should not be interpreted only as a characteristic of K-means, but also as a consequence of the spatial distribution of sensors produced by the optimization process.

The results obtained are consistent with studies that highlight the advantages of hierarchical architectures to improve the organization, connectivity, and efficiency of the WSN [21,144]. It was demonstrated that an adaptive hierarchical architecture can achieve high levels of coverage with a reduction in the number of CHs [21], while benefits from the combination of optimized positioning and spatial clustering were shown in [144]. The approach proposed here adds an explicit environmental dimension to these strategies, since the hierarchical organization is built on a network initially oriented by hazard.

The need for multi-hop routing results directly from the spatial scale of the network. The average distance between the sensors and their respective CHs (1425–1494 m) is about six times the 250 m communication range, and the median distance between neighboring sensors (559 m) is more than twice that range. Direct communication between sensors and CHs would therefore not be compatible with the adopted range, and even multi-hop routing over sensors alone would leave about 94% of the sensors disconnected. Connectivity was only achieved by adding about two relay nodes per sensor.

This result exposes a tension between coverage and connectivity that is inherent to coverage-driven placement. The greedy heuristic spreads sensors to avoid overlapping coverage, whereas a short communication range requires closely spaced nodes. Under the conservative 250 m assumption, relays more than doubled the number of deployed nodes (1748 to 1763 nodes in total for 589 sensors) and accounted for about two thirds of the intra-cluster energy, which affects deployment cost as much as energy consumption. Connectivity-aware placement formulations, or a less conservative communication range supported by field measurements, are therefore natural extensions of this work.

The use of multiple hops is consistent with the principles of hierarchical WSN architectures, in which aggregation and distributed routing are used to avoid direct long-distance transmissions. LEACH [113], for example, introduced a CH-based organization to reduce the need for direct communication with the sink, while PEGASIS [145] explored sequential routing structures to reduce transmission distances. Subsequent studies continue to demonstrate the importance of the integration between clustering, routing, and topological optimization in WSN performance [21,55,98].

However, the results also show that the use of multi-hop should not be automatically interpreted as synonymous with lower energy consumption. Although several short links can replace a long transmission, each additional hop involves transmission and reception operations. The energy benefit therefore depends on the combination of link length, number of hops, amount of data transmitted, and energy model parameters. In the present study, routes comprised on average 11.4 to 12.2 hops, so the reception and electronics costs of each hop represent a non-negligible share of the intra-cluster energy.

The MST-based inter-cluster structure complements this organization by providing a connected topology between the clusters with the minimum number of links required for a tree. The use of 27, 28, and 29 connections in the 28, 29, and 30 cluster configurations, respectively, is a direct consequence of this MST property. The structure therefore allows for organizing communication between clusters without introducing redundant cycles in the considered topology.

It is important to highlight, however, that increasing the number of clusters did not produce a monotonic reduction in the average distance of the inter-cluster links. The values obtained for MST were 4098 m, 4158 m, and 3977 m for 28, 29, and 30 clusters, respectively. This behavior demonstrates that the relationship between the number of clusters and the cost of inter-cluster communication depends on the spatial configuration of the clusters and not just on the number of clusters.

Consequently, the results do not support a simplified interpretation according to which increasing the number of clusters would necessarily lead to an overall reduction in communication distances. Increasing (k) reduces the average size of the clusters and tends to bring the sensors closer to their respective CHs but simultaneously increases the number of CHs that need to be interconnected. The overall performance results from the balance between these two effects.

The observed stability in the sink’s location among the evaluated configurations constitutes another indicator of the solution’s spatial consistency. The selection of the same cluster as a sink in the configurations with 29 and 30 clusters, and a cluster located in a similar central region in the configuration with 28 clusters, suggests that small changes in the number of clusters did not substantially modify the overall spatial structure of the clusters.

This characteristic is relevant because the sink was not previously defined in a fixed location but selected from the distribution of clusters. The strategy allows adapting the collection point to the network geometry, being particularly suitable for environmentally heterogeneous areas where the distribution of sensors does not necessarily present a regular configuration.

The central location of the sink favors a shallow inter-cluster tree (on average 3.9 to 5.2 hops between each CH and the sink), but under the aggregation assumption adopted, it does not affect the estimated energy consumption, which depends on the MST links and not on the choice of root. Its effect on energy would only become apparent if the traffic forwarded by each CH were modelled without aggregation.

Furthermore, the use of a single sink constitutes a potential limitation for networks intended to operate for extended periods. The concentration of traffic at a collection point can increase the load on CHs located near the sink and contribute to the development of the so-called energy hole problem [146]. The present analysis does not allow quantifying this effect, since the consumption of one monitoring round was evaluated and not the temporal evolution of the energy level of the nodes. The analysis of the network’s lifespan and the spatial distribution of the energy load should, therefore, constitute a natural extension of this work.

The energy results show only small differences between the cluster configurations. Consumption was 347.36 J, 349.59 J and 343.20 J per round for 28, 29 and 30 clusters, respectively. In the reference run, the configuration with 30 clusters presented the lowest energy consumption, 1.2% below the configuration with 28 clusters and 1.8% below the configuration with 29 clusters, while the configuration with 29 clusters presented the highest value. Given the variation observed across K-means seeds (Section 4.10.1), these differences are interpreted descriptively rather than as evidence that one configuration is superior.

The intra-cluster component decreased between 28 and 29 clusters (from 143.20 J to 133.66 J), in parallel with the decrease in the average sensor-CH distance (from 1494 m to 1440 m) and increased slightly for 30 clusters (136.56 J; 1425 m). Considering the first-order radio model in [113], shorter intra-cluster distances reduce the number of hops and relays required, which explains part of this behavior, although the intra-cluster energy also depends on how the sensors of each cluster are arranged around their CH.

However, the inter-cluster component, which represents about 60% of the total consumption, did not follow the same trend. It was highest for 29 clusters (215.94 J), the configuration with the longest MST (116.44 km), which offset the intra-cluster gain and made 29 clusters the most energy-demanding configuration. The lower consumption observed for 30 clusters should thus be understood as a result of the interaction between the intra-cluster and inter-cluster components, and not as an exclusive consequence of the reduction in the sensor-CH distance.

This result reinforces the existence of a trade-off between cluster granularity and communication costs. A reduced number of clusters implies larger clusters and, consequently, greater average distances between sensors and CHs. On the other hand, an excessively high number of clusters would increase the number of CHs and could raise the cost associated with inter-cluster communications. The existence of this trade-off means that the configuration with 30 clusters can only be described as the lowest-consumption configuration in the reference run, not as an optimum, and not as a demonstration that any additional increase in (k) would necessarily lead to an energy improvement.

Maintaining the coverage of priority cells and network connectivity in all three configurations adds an important dimension to the interpretation of the results. In the set of configurations analyzed, the differences in energy consumption were not accompanied by a loss of priority spatial coverage or of connectivity. This result is particularly relevant for fire detection applications, where a reduction in energy consumption is only operationally useful if it does not compromise the ability to monitor priority areas.

The comparison between scenarios clarifies the contribution of the combined procedure relative to random placement. With the same number of sensing nodes, random placement covered only 45.9% of the high- and very-high-hazard cells in the draw with seed = 42 (45.3 ± 1.5% across 100 placements), while consuming 2.9% less energy per round in that draw; over all placements and clustering seeds, the mean total energy of the two scenarios was practically identical (339.8 ± 11.9 J versus 339.9 ± 7.2 J). The benefit of the proposed approach is therefore not a lower absolute energy consumption, but a much more effective use of the sensing effort: the reference configuration required about half the energy per covered priority cell (117 mJ versus 247 mJ). The slightly lower consumption of the random network results from a more regular spatial arrangement of its CHs, which shortened the longest links of the idealized inter-cluster MST, and illustrates that energy and coverage must be assessed jointly. The supplementary analyses of Section 4.10.2 indicate that 15.8 of the 54.7 percentage points of the coverage gain are attributable to coverage optimization alone and 38.9 percentage points (about 71%) to hazard prioritization; the gain is attributed to the hazard map only to that extent. Under the link-constrained variant (Section 4.10.3), the reference scenario also consumed 5.6% less energy per round than the random control.

The alternative scenario shows the cost of a less restrictive hazard threshold. Including Class 3 cells more than doubled the priority set (from 2995 to 6307 cells) and required 79% more sensors, 51% more relays and, with the cluster count fixed at k = 29, 71% more energy per round. This energy increase is conditional on the fixed cluster count, which nearly doubled the average cluster size; with k = 53 it was 21% (Section 4.10.4). The choice of the threshold is therefore a planning decision that balances the extent of the monitored area against deployment and operating costs, and the proposed method allows this trade-off to be quantified before installation.

Some limitations should be noted. The uncertainty analysis (Section 4.10.1) covers the variability due to random placement and K-means initialization, but not the uncertainty of the hazard map or of the radio parameters. The number of relays depends strongly on the 250 m communication range, adopted as a conservative assumption, and on the relay placement heuristic, which does not guarantee a minimum number of relays. The inter-cluster links of the idealized MST, up to about 9.4 km long, are not substantiated as feasible for identical sensor nodes in the study area; the idealized variant establishes mathematical connectivity only, and the link-constrained variant (Section 4.10.3) should be used for any estimate of deployment size or inter-cluster energy. Finally, the comparison with random placement is made at an equal number of sensing nodes and not at equal total deployment cost, since relay counts differ between scenarios. These aspects should be considered in operational planning.

## 6. Conclusions

In summary, the results indicate that the use of official hazard mapping as a structuring element in the planning of Wireless Sensor Networks allows for the effective integration of spatial analysis, combinatorial optimization, and hierarchical communication organization. The proposed methodology articulates the identification of the most hazardous areas, the optimization of sensor positioning through MCP, the formation of clusters, and the structuring of communication through relay-assisted multi-hop routing and a Minimum Spanning Tree. In the Serra de São Mamede Natural Park, the greedy heuristic placed 589 sensors covering all 2995 high- and very-high-hazard cells, more than twice the coverage achieved by random deployments with the same number of sensing nodes for a similar energy budget; this gain reflects the combined effect of hazard prioritization and coverage optimization. Ensuring connectivity under a conservative 250 m range required about two relay nodes per sensor, and the estimated consumption varied little among 28–30 clusters (≤1.8%). The inter-cluster MST without a range limit is an idealized connected graph; under the same 250 m link constraint, the inter-cluster layer required about 450 additional relays and its energy fell from about 208 J to 11 J per round.

The main contribution of the study, therefore, consists of an integrated and reproducible approach for planning risk-oriented networks, with potential application in intelligent early detection systems for forest fires. The methodology could be further developed by incorporating dynamic factors, such as weather conditions, temporal variations in risk, and actual energy consumption, aiming for a more adaptive optimization closer to operational applications. Connectivity-aware placement and field measurements of LoRa range in forest environments are also priorities for future work.

## Figures and Tables

**Table 1:** Cluster statistics in the reference scenario (k = 28, 29 and 30) and in the alternative and control scenarios (k = 29). Values are mean ± standard deviation, with the range in parentheses.

Scenario	k	Sensors	Sensors per Cluster	Sensor Distance → CH
(i) Reference	28	589	21.0 ± 5.6 (8–30)	1494 ± 738 m
(i) Reference	29	589	20.3 ± 6.6 (7–32)	1440 ± 697 m
(i) Reference	30	589	19.6 ± 5.8 (8–28)	1425 ± 688 m
(ii) Alternative	29	1054	36.3 ± 4.3 (27–48)	1649 ± 680 m
(iii) Control	29	589	20.3 ± 4.1 (12–30)	1585 ± 750 m

**Table 2:** Intra-cluster connectivity in the three scenarios.

Scenario	k	Relays	Relays per Sensor	Total Nodes	Hops per Sensor, Mean (max)	Relay Share of E Intra
(i) Reference	28	1174	1.99	1763	12.2 (45)	66.2%
(i) Reference	29	1159	1.97	1748	11.4 (34)	65.3%
(i) Reference	30	1169	1.98	1758	11.7 (48)	66.7%
(ii) Alternative	29	1751	1.66	2805	18.0 (71)	63.3%
(iii) Control	29	1208	2.05	1797	12.7 (51)	66.2%

**Table 3:** Inter-cluster structure (MST) and sink in the three scenarios.

Scenario	k	MST Edges	Mean Link Length (m)	Total Length (km)	Longest Link (m)	Sink CH (Position)	CH → Sink Hops, Mean (Max)
(i) Reference	28	27	4098 ± 1451	110.65	9361	8 (65, 108)	3.9 (8)
(i) Reference	29	28	4158 ± 1409	116.44	9361	14 (65, 111)	4.6 (10)
(i) Reference	30	29	3977 ± 1412	115.34	9361	14 (62, 109)	5.2 (9)
(ii) Alternative	29	28	4211 ± 570	117.89	6486	28 (64, 100)	5.5 (11)
(iii) Control	29	28	4094 ± 809	114.64	6605	6 (57, 123)	6.5 (12)

**Table 4:** Energy consumption per data-collection round in the three scenarios (E Relays is the part of E Intra consumed by relay nodes; the energy per covered cell refers to Class 4–5 cells).

Scenario	k	Coverage of Classes 4–5	E Intra (J)	E Inter (J)	E Relays (J)	E Total (J)	Energy per Sensor (mJ)	Energy per Covered Cell (mJ)
(i) Reference	28	100%	143.20	204.16	94.81	347.36	590	116
(i) Reference	29	100%	133.66	215.94	87.33	349.59	594	117
(i) Reference	30	100%	136.56	206.64	91.11	343.20	583	115
(ii) Alternative	29	100%	394.06	202.21	249.37	596.27	566	199
(iii) Control	29	45.9%	144.31	195.09	95.52	339.40	576	247

**Table 5:** Stability of the main metrics across independent seeds. Values are mean ± SD, with the 2.5–97.5 percentile interval in parentheses.

Scenario	k	Source of Variation	Runs	Coverage of Classes 4–5 (%)	Relays	E Total (J)	Energy per Covered Cell (mJ)
(i) Reference	28	Clustering	30	100	1169 ± 7 (1156–1182)	346.4 ± 7.0 (334.8–357.2)	115.7 ± 2.3 (111.8–119.3)
(i) Reference	29	Clustering	30	100	1164 ± 6 (1154–1174)	339.9 ± 7.2 (327.6–355.1)	113.5 ± 2.4 (109.4–118.6)
(i) Reference	30	Clustering	30	100	1163 ± 8 (1147–1176)	338.8 ± 7.0 (329.8–351.0)	113.1 ± 2.3 (110.1–117.2)
(iii) Control	29	Placement + clustering	100 × 30	45.3 ± 1.5 (42.6–48.0)	1200 ± 27 (1141–1260)	339.8 ± 11.9 (316.8–363.8)	250.8 ± 12.2 (228.5–274.3)
(iii) Control: share of variance due to placement	29	—	—	100%	87.5%	51.5%	74.7%

**Table 6:** Coverage and network metrics under a common feasible domain and for the hazard-blind control (k = 29). All scenarios use 589 sensing nodes. Values are mean ± SD over 30 K-means seeds (and over 100 placements for (iii) and 20 tie-breaking seeds for (iv)).

Scenario	Feasible Positions	Hazard Information	Sensors	Coverage of Classes 4–5	Relays	E Total (J)	Energy per Covered Cell (mJ)
(i) Reference	All grid cells	Yes	589	100%	1164 ± 6	339.9 ± 7.2	113.5 ± 2.4
(i-c) Reference, common domain	Classified cells	Yes	589	100%	1162 ± 7	340.4 ± 8.3	113.7 ± 2.8
(iv) Hazard-blind optimized	Classified cells	No	589	61.1 ± 1.1%	1626 ± 14	363.1 ± 10.3	198.5 ± 6.6
(iii) Random	Classified cells	No	589	45.3 ± 1.5%	1200 ± 27	339.8 ± 11.9	250.8 ± 12.2

**Table 7:** Inter-cluster structure without a range limit (idealized MST) and under the 250 m link constraint. Values are mean ± SD over 30 K-means seeds (and 100 placements for the control). Total nodes = sensors + intra-cluster relays + inter-cluster relays; under the link constraint, no link exceeds 250 m.

Scenario	k	Inter-Cluster Relays	Total Nodes	E Inter, Idealized (J)	E Inter, Constrained (J)	E Total, Idealized (J)	E Total, Constrained (J)
(i) Reference	28	443 ± 7	2201	210.7 ± 6.2	11.15 ± 0.18	346.4 ± 7.0	146.9 ± 4.4
(i) Reference	29	447 ± 8	2200	207.9 ± 6.1	11.28 ± 0.19	339.9 ± 7.2	143.3 ± 5.2
(i) Reference	30	455 ± 7	2206	208.7 ± 6.0	11.49 ± 0.16	338.8 ± 7.0	141.6 ± 4.9
(ii) Alternative	29	464 ± 6	3286	206.0 ± 4.5	11.72 ± 0.14	555.3 ± 17.1	361.1 ± 16.9
(iii) Control	29	454 ± 10	2244	199.6 ± 8.5	11.50 ± 0.26	339.8 ± 11.9	151.8 ± 7.3

**Table 8:** Alternative scenario (Classes 3–5) with the cluster count of the reference scenario (k = 29) and with approximately 20 sensors per cluster (k = 53). The first row is the single run of Table 1, Table 2, Table 3 and Table 4 (1054 sensors); the other rows are means ± SD over 30 K-means seeds of the re-implementation (1057 sensors). Increases are relative to the reference scenario with k = 29 under the same procedure.

Scenario	k	Sensors per Cluster	Relays	Hops per Sensor, Mean (Max)	E Intra (J)	E Inter (J)	E Total (J)	Increase, Idealized MST	Increase, Link-Constrained
(ii) Single run	29	36.3	1751	18.0 (71)	394.06	202.21	596.27	+70.6%	—
(ii) 30 seeds	29	36.4	1765 ± 6	16.5 (59)	349.4 ± 16.9	206.0 ± 4.5	555.3 ± 17.1	+63.4%	+152%
(ii) 30 seeds	53	19.9	1735 ± 7	10.7 (40)	219.3 ± 8.6	192.1 ± 5.0	411.3 ± 9.7	+21.0%	+64%

## Data Availability

The official fire hazard cartography used in this study is publicly available from the Instituto da Conservação da Natureza e das Florestas (ICNF, Portugal). The processed hazard matrices, sensor-position outputs, and simulation code generated during this study are available from the corresponding author upon reasonable request.

## Abbreviations

The following abbreviations are used in this manuscript:

WSN	Wireless Sensor Network
ICNF	Institute for Nature Conservation and Forests
PNSSM	Parque Natural da Serra de São Mamede
MCP	Maximum Coverage Problem
CH	Cluster Head
MST	Minimum Spanning Tree
GIS	Geographic Information System
LEACH	Low-Energy Adaptive Clustering Hierarchy
PSO	Particle Swarm Optimization
LoRa	Long Range (low-power wide-area network technology)
